# The Role of Vesicular Glutamate Transporter Type 3 in Social Behavior, with a Focus on the Median Raphe Region

**DOI:** 10.1523/ENEURO.0332-23.2024

**Published:** 2024-06-03

**Authors:** Csilla Lea Fazekas, Bibiána Török, Pedro Correia, Tiago Chaves, Manon Bellardie, Eszter Sipos, Hanga Réka Horváth, Balázs Gaszner, Fanni Dóra, Árpád Dobolyi, Dóra Zelena

**Affiliations:** ^1^Institute of Physiology, Medical School, Centre for Neuroscience, Szentágothai Research Centre, University of Pécs, Pécs 7624, Hungary; ^2^Institute of Experimental Medicine, Eötvös Loránd Research Network, Budapest 1084, Hungary; ^3^János Szentágothai Doctoral School of Neurosciences, Semmelweis University, Budapest 1085, Hungary; ^4^Department of Anatomy, Medical School, Centre for Neuroscience, Szentágothai Research Centre, University of Pécs, Pécs 7624, Hungary; ^5^Human Brain Bank and Microdissection Laboratory, Semmelweis University, Budapest 1085, Hungary; ^6^Laboratory of Neuromorphology, Department of Anatomy, Histology and Embryology, Semmelweis University, Budapest 1085, Hungary; ^7^Laboratory of Molecular and Systems Neurobiology, Department of Physiology and Neurobiology, Eötvös Loránd University, Budapest 1117, Hungary

**Keywords:** chemogenetic, median raphe, social behavior, VGluT3, VGluT3-Cre mice, VGluT3 knock-out mice

## Abstract

Social behavior is important for our well-being, and its dysfunctions impact several pathological conditions. Although the involvement of glutamate is undeniable, the relevance of vesicular glutamate transporter type 3 (VGluT3), a specific vesicular transporter, in the control of social behavior is not sufficiently explored. Since midbrain median raphe region (MRR) is implicated in social behavior and the nucleus contains high amount of VGluT3+ neurons, we compared the behavior of male VGluT3 knock-out (KO) and VGluT3-Cre mice, the latter after chemogenetic MRR-VGluT3 manipulation. Appropriate control groups were included. Behavioral test battery was used for social behavior (sociability, social discrimination, social interaction, resident intruder test) and possible confounding factors (open field, elevated plus maze, Y-maze tests). Neuronal activation was studied by c-Fos immunohistochemistry. Human relevance was confirmed by VGluT3 gene expression in relevant human brainstem areas. VGluT3 KO mice exhibited increased anxiety, social interest, but also aggressive behavior in anxiogenic environment and impaired social memory. For KO animals, social interaction induced lower cell activation in the anterior cingulate, infralimbic cortex, and medial septum. In turn, excitation of MRR-VGluT3+ neurons was anxiolytic. Inhibition increased social interest 24 h later but decreased mobility and social behavior in aggressive context. Chemogenetic activation increased the number of c-Fos+ neurons only in the MRR. We confirmed the increased anxiety-like behavior and impaired memory of VGluT3 KO strain and revealed increased, but inadequate, social behavior. MRR-VGluT3 neurons regulated mobility and social and anxiety-like behavior in a context-dependent manner. The presence of VGluT3 mRNA on corresponding human brain areas suggests clinical relevance.

## Significance Statement

The control of social behavior is complex, and the role of numerous subcortical areas in it is still poorly understood. In our experiments, we investigated a subpopulation of glutamatergic neurons that express the vesicular glutamate transporter type 3 (VGluT3), especially those found in the median raphe region (MRR) in the control of social behavior, locomotion, anxiety, and memory. In mice completely lacking VGluT3, the highly anxious behavior, alterations in locomotion, and impaired memory were confirmed, and increased social interest and inadequate aggressive behavior were also revealed. MRR-VGluT3 neurons proved to be at least partially responsible for the locomotor and anxiety-like behavior, as well as social interest. Lastly, translational relevance was proved by detection of the VGluT3 gene expression in the human brainstem.

## Introduction

Vesicular glutamate transporter type 3 (VGluT3) is the latest transporter discovered from the solute carrier (SLC) 17 protein family (Fremeau et al., 2002), and thus, its role in behavior is still largely unknown. Homozygous VGluT3 knock-out (KO) mice are viable (Horvath et al., 2018) and their behavioral characterization is ongoing (Seal et al., 2008; Amilhon et al., 2010; Divito et al., 2015; Balazsfi et al., 2016, 2018a; Fazekas et al., 2019). However, detailed investigation of its contribution to social behavior is still missing, despite the importance of these interactions in everyday life as well as its disturbances in psychopathologies.

Numerous brain regions contain VGluT3-positive neurons, such as the hippocampus, cortex, and median raphe region (MRR) in the midbrain. The MRR is known as one of the serotoninergic nuclei, which provides serotoninergic innervation to the forebrain (Dahlstrom and Fuxe, 1964; Vertes et al., 1999). However, only 5.742% of the MRR neurons have been shown to express serotonin (5-HT; Sos et al., 2017). The rest of the cells express numerous other neurotransmitters and neuromodulators, such as GABA (Calizo et al., 2011; Sos et al., 2017), dopamine (Jahanshahi et al., 2013), and glutamate, characterized by VGluT2 (Szonyi et al., 2019) or VGluT3 (Fremeau et al., 2002; Herzog et al., 2004; Amilhon et al., 2010; Sos et al., 2017). According to our current knowledge, ∼10.651% of the MRR cells contain VGluT3 as a neuronal marker, while ∼3.603% of the MRR cells show serotonin and VGluT3 coexpression as well (Sos et al., 2017). These VGluT3-positive cells target key areas such as the medial prefrontal cortex (mPFC), hippocampus, thalamus, hypothalamus, and dorsal raphe (Jackson et al., 2009; Szonyi et al., 2016). Numerous studies have investigated the role of MRR in behavior (Wang et al., 2015; Bland et al., 2016; Balazsfi et al., 2017; Turcotte-Cardin et al., 2019), but they were solely concentrating on its serotonergic content. However, it was demonstrated that at least in certain areas (e.g., mPFC, hippocampus, medial septum), functional glutamate receptors are expressed in the postsynaptic sites of these MRR-VGluT3–positive projections (Szonyi et al., 2016). The optogenetic stimulation of the whole MRR decreased aggressive behavior in a phasic manner in mice, which was accompanied by increased serotoninergic, GABAergic, and glutamatergic neurotransmitter levels in the mPFC (Balazsfi et al., 2018b). While it is established that MRR regulates social behavior, the specific role of its VGluT3+ neurons is still unknown.

Our aim was to characterize the role of VGluT3 in social behavior. With the usage of VGluT3 knock-out (KO) mice, the global role of this transporter could be revealed; however, life-long, constitutive lack of a gene product could result in compensatory mechanisms, ultimately masking behavioral effects. The chemogenetic manipulation of MRR-VGluT3 neurons would shed new light on the role of this specific subpopulation giving the opportunity for both stimulatory and inhibitory short-term interventions. The comparison of the two models would provide deeper insight on the role of VGluT3.

## Materials and Methods

### Animals

**Figure 1.**
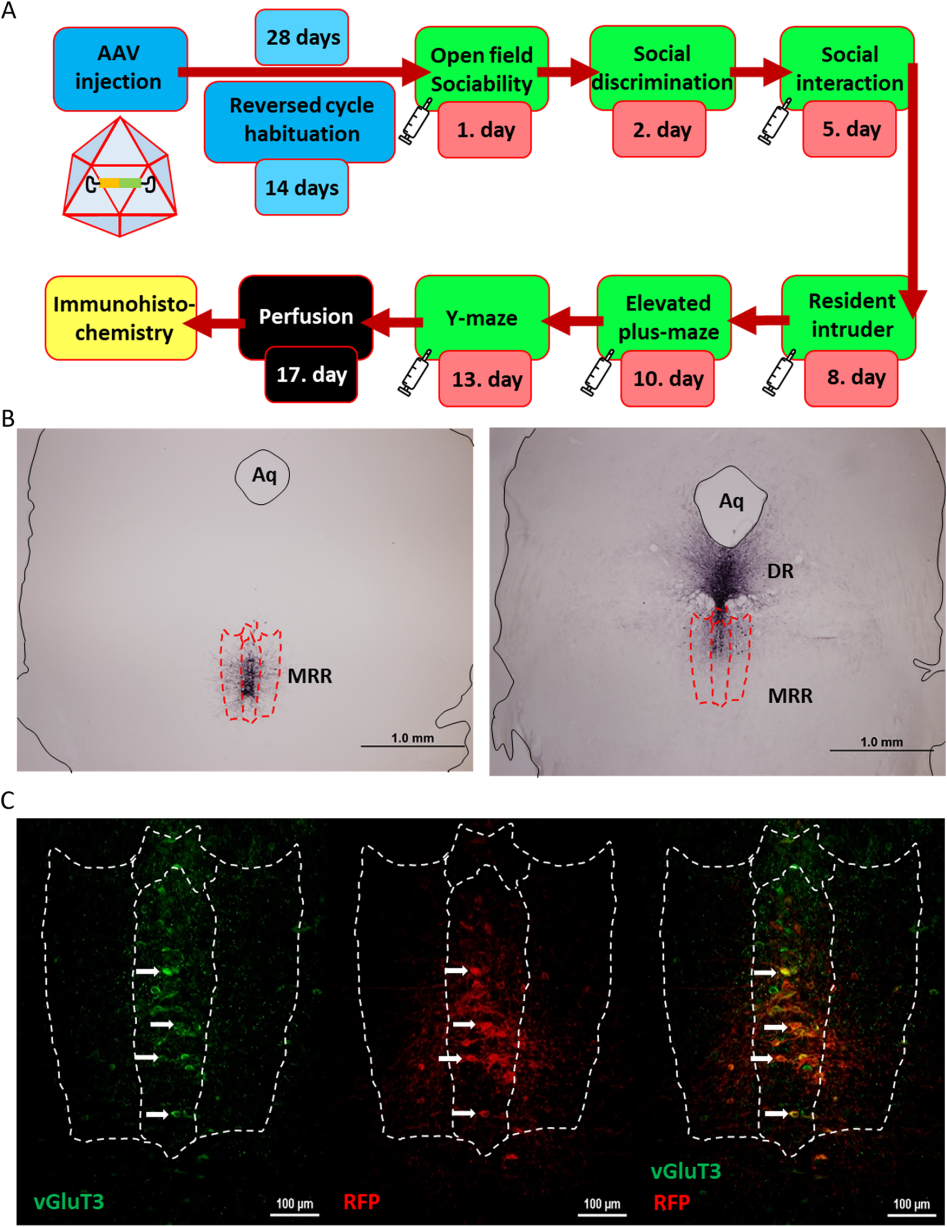
Timeline of the experimental protocols and immunohistochemical validation. ***A***, Schematic timeline of the behavioral test battery. In case of VGluT3-Cre mice, experiments started 4 weeks after the stereotaxic surgery—which included 2 weeks of habituation to “reversed” day/night cycle—in order to ensure viral expression of DREADDs. Syringes indicate the occasions when VGluT3-Cre mice were injected intraperitoneally with CNO (1 mg/ml/kg) 30 min before the start of the experiment. Two to 3 d of break were kept between the experiments in order to eliminate the carry-over effect of CNO. WT and VGluT3 KO mice went under the same procedure, except for the surgery and CNO administration. ***B***, Example of proper (left) and improper (right) viral expression in VGluT3-Cre mice. After the experiments, brains were collected from the animals, and Ni-DAB staining was done on midbrain slices against the RFP encoded in the AAV. Only the data of those animals that had proper viral expression in the MRR were included in the statistical analysis. On the right, there is an example of unacceptable staining in the DR. ***C***, Representative figure of viral expression specificity in the MRR. Double fluorescent immunohistochemistry was done of midbrain slices against VGluT3 (Alexa-488, green) and RFP (Alexa-594, red) in control virus injected VGluT3-Cre mice. White arrows indicate example neurons where both markers were found. Scale bar, 100 μm. AAV, adeno-associated virus vector; Aq, Aqueduct cerebri; CNO, clozapine-*N*-oxide; DR, dorsal raphe; DREADD, designer receptor exclusively activated by designer drugs; KO, knock-out; MRR, median raphe region; Ni-DAB, nickel-3.3′-diaminobenzidine; RFP, red fluorescent protein; VGluT3, vesicular glutamate transporter 3; WT, wild-type.

All mice (C57BL/6J background) were obtained from the local colonies of the Institute of Experimental Medicine, Budapest, Hungary. VGluT3 wild-type (WT) and KO mice were bred in heterozygous mating pairs. Their genotype was determined by PCR from a small tail sample collected from 2- to 3-day-old animals. VGluT3-Cre mice originated from homozygous mating. During the test battery adult male mice (14–15-week-old) were housed in Makrolon cages (40 cm × 25 cm × 26 cm) under a “reversed” 12 h light/dark cycle (lights on at 7 P.M., 21 ± 1°C, 50–60% humidity), with food (standard mice chow, Charles River) and tap water available *ad libitum*. Thirty minutes before the start of the first experiment, the animals were separated to increase social interest during subsequent testing. The experiments were conducted between 9 and 13 h, during the subjective dark period of the mice. The KO-WT and VGluT3-Cre animals were tested in different series with minor differences in the protocol (Fig. 1*A*):

1. Series 1: WT (*N* = 10) and VGluT3 KO (*N* = 11) animals were compared.
2. Series 2: VGluT3-Cre animals went under stereotaxic surgery. Three subgroups were formed based on the injected viral vectors containing “designer receptors exclusively activated by designer drug” (DREADD) sequences: control (*N* = 8), excitatory (*N* = 13), and inhibitory (*N* = 15).

c-Fos activation was studied in a different batch of animals from those used in the behavioral studies. Wild-type (*N* = 5) and VGluT3 KO (*N* = 8) mice were perfused 90 min after a social interaction test (SIT). In the case of VGluT3-Cre mice (*N*_control _= 6; *N*_excitatory _= 3), separate animals were injected intraperitoneal (i.p.) with clozapine-*N*-oxide (CNO, ligand of DREADD; CAS No.: 34233-69-7, Tocris Bioscience, 1 mg/kg/10 ml saline) and perfused 30 (time for CNO to take effect) + 90 min (c-Fos expression peak) after injection.

All tests were approved by the local committee of animal health and care (PEI/001/33-4/2013, PE/EA/254-7/2019) and performed according to the European Communities Council Directive recommendations for the care and use of laboratory animals (2010/63/EU).

### Surgery

Anesthetized [0.1 ml/10 g mixture of 0.5 ml ketamine (Produlab Pharma), 0.1 ml xylazine (Produlab Pharma), and 2.4 ml saline (KabiPac)] VGluT3-Cre mice were stereotaxically (David Kopf Instruments) injected with an adeno-associated virus vector (AAV8) containing DREADD and/or red fluorescent protein (RFP) sequences between two loxP loci (20 nl; Addgene) into the MRR (AP: −4.1 mm; L: 0 mm; DV: 4.6 mm from bregma; Balazsfi et al., 2017, 2018b; Fazekas et al., 2021; Chaves et al., 2022). Three groups were formed: control (only RFP; #50459); excitatory (*G_q_* stimulation; #44361), and inhibitory (*G_i_* inhibition; #44362). Animals had 28 d to recover, of which they adapted to the reverse cycle for 14 d.

### Experimental design

Thirty minutes before the experiments (except for social discrimination test; SDT), VGluT3-Cre animals were injected intraperitoneally with CNO (Chaves et al., 2022). There were 48–72 h intervals between behavior tests (except for SDT) to ensure the clearance of previously injected CNO (Fig. 1*A*). For comparability the timing was the same in both series.

The experimental room was dark, only lit by infrared light [except for SIT and elevated plus maze (EPM)]. The experiments were recorded and analyzed later by computer-based event recorders (H77; Solomon Coder (https://solomoncoder.com); Noldus EthoVision). The test apparatuses were cleaned with 20% ethanol between animals except for SIT and RIT (with bedding).

In the end, the animals were anaesthetized by ketamine–xylazine and fixed by transcardial perfusion with phosphate-buffered saline (PBS; Molar Chemicals) and 4% paraformaldehyde (PFA; Molar Chemicals). After 24 h postfixation in PFA, the solution was changed to azide-PBS (Na-azide from Sigma-Aldrich). Before cutting, the brains were immersed in 20% glucose (Reanal) azide-PBS for at least 24 h. Thirty-micrometer slices were cut with sledge microtome and stored in cryoprotectant (all components from Molar Chemicals) at −20°C. Immunohistochemistry was done for RFP and VGluT3 in order to validate the virus expression (Fig. 1*B*) and cell specificity (Fig. 1*C*). Staining for c-Fos was conducted to explore neuronal activity.

#### Open field

**Figure 2.**
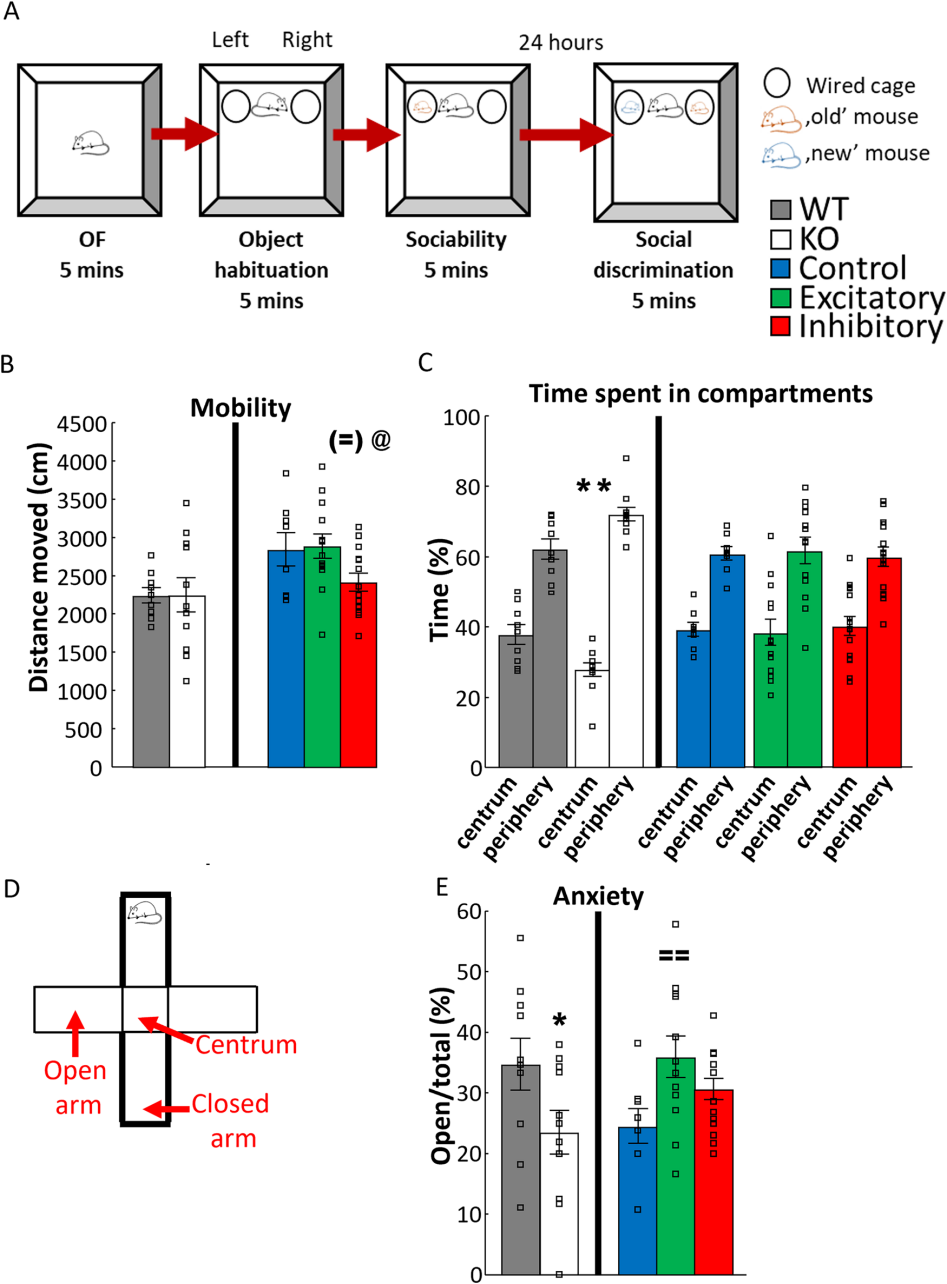
Results of experiments on mobility and anxiety-like behavior. ***A***, Schematic figure of sociability test, which started with a 5 min of OF test for mobility and anxiety-like behavior. Right after that, 5 min of object habituation followed when two identical wired cages were put into the arena. Right after that, one unfamiliar, juvenile conspecific was put under one of the wired cages for sociability phase. One day later, a 5 min SDT was conducted, during which the already familiar and a new unfamiliar conspecific were placed under the wired cages with interchanged positions compared with the previous day. ***B***, Distance moved during OF. In dark, there was no difference between WT and VGluT3 KO mice (*t* test, *N*_WT _= 9; *N*_KO _= 11). On the other hand, inhibition of VGluT3+ neurons in the MRR resulted in decreased mobility (one-way ANOVA; *N*_control _= 8; *N*_excitatory _= 13; *N*_inhibitory _= 14). ***C***, Percentage of time in different zones during OF. Increased anxiety-like behavior (repeated-measures ANOVA) in VGluT3 KO mice is reflected by decreased time spent in the centrum (75% of the arena floor). ***D***, Schematic representation of EPM apparatus. EPM consists of two closed arms and two open arms connected by a centrum area, elevated 67 cm above the ground. ***E***, Open/total (%) is a locomotion independent parameter that reflects anxiety-like behavior on EPM. Increased anxiety-like behavior is reflected by decreased open/total (%) in VGluT3 KO mice (*t* test; *N*_WT _= 10; *N*_KO _= 11). On the other hand, the excitation of MRR-VGluT3+ neurons elicited anxiolytic effect (one-way ANOVA; *N*_control _= 8; *N*_excitatory _= 15; *N*_inhibitory _= 15). Data are expressed as average ± SEM. Empty squares represent individual values. For more details see Extended Data Tables to Figure 2. EPM, elevated plus maze test; KO, knock-out; MRR, median raphe region; OF, open field test; VGluT3, vesicular glutamate transporter type 3; WT, wild-type. **p < 0.01 versus WT; ^(=)^*p* = 0.08, ^==^*p* < 0.01 versus control; ^@^*p* < 0.05 versus excitatory.

Open field (OF) measures the locomotor activity and anxiety-like behavior of mice. Animals were put into an empty white plastic box (40 cm × 36 cm × 15 cm; Fig. 2*A*). Distance traveled, frequency, and time spent in periphery and centrum (75% of the arena) were analyzed by the Noldus EthoVision XT 13.

#### Sociability

Right after OF, two identical wired cages (with identical weights on them) were placed into the empty plastic boxes (object habituation phase; Fig. 2*A*). Based on their location, they were either labeled as “left” or “right.” After 5 min, an unknown, smaller, juvenile male conspecific was placed under one of the cages for 5 min (sociability phase).

Social preference index (SI) was calculated as follows:

$$\mathrm{SI}=\frac{t_{\mathrm{mouse}}}{t_{\mathrm{mouse}}+t_{\mathrm{cage}}}\times 100,$$

where *t*_mouse_ is the time spent sniffing the stimulus mouse; *t*_cage_ is the time spent sniffing the empty cage.

Any other type of behavior was labeled as “other.”

#### Social discrimination

Twenty-four hours after sociability test, SDT was conducted. The experimental setting was identical to that of sociability (Fig. 2*A*). Under each wired cage, a conspecific was placed for 5 min. One was used during sociability (“old”), while the other one was unknown (“new”). Here CNO was not injected; thus, the effect on memory consolidation and not on recall was tested.

Social discrimination index (SD) was calculated as follows:

$$\mathrm{SD}=\frac{t_{“\mathrm{old}”}-t_{“\mathrm{new}”}}{t_{“\mathrm{old}”}+t_{“\mathrm{new}”}}\times 100,$$

where *t*_“old”_ is the time spent sniffing the familiar stimulus mouse; *t*_“new”_ is the time spent sniffing the unfamiliar stimulus mouse.

#### Social interaction

**Figure 3.**
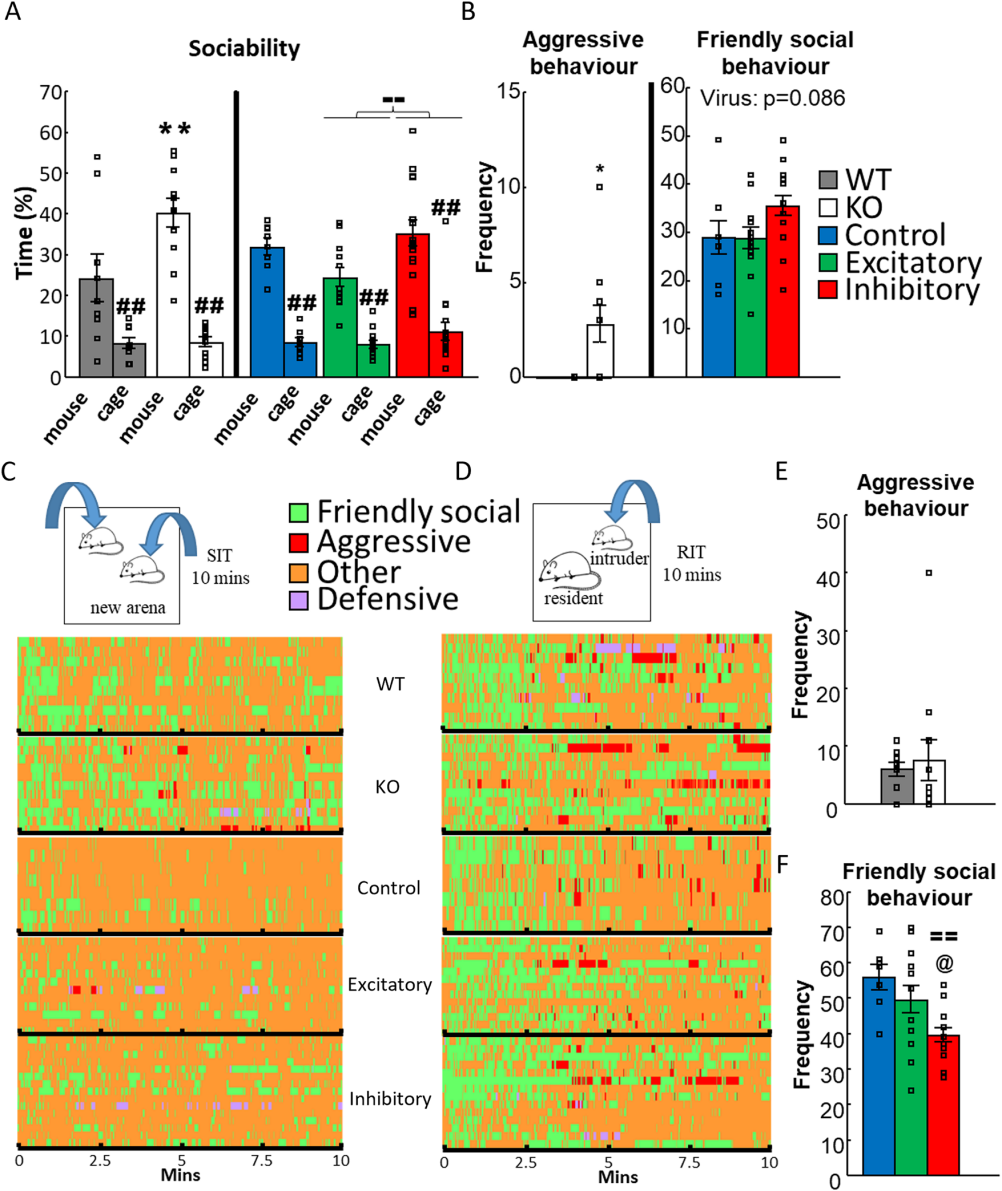
Results of experiments on social behavior. ***A***, Percentage of time spent with empty cages and the cage containing an unfamiliar, juvenile conspecific. Both WT and VGluT3 KO mice preferred the social stimuli compared with the inanimate object, but KO animals spent more time with the conspecific (repeated-measures ANOVA; *N*_WT _= 10; *N*_KO _= 11). Similarly, all animals in the VGluT3-Cre groups preferred the social stimuli, but the excitatory group had decreased interest compared with the inhibitory group (repeated-measures ANOVA; *N*_control _= 8; *N*_excitatory _= 11; *N*_inhibitory _= 15). ***B***, Main differences between groups in SIT. VGluT3 KO mice exhibited more aggressive behavior compared with the WT animals (*t* test; *N*_WT _= 10; *N*_KO _= 11). On the other hand, friendly social behavior was only marginally changed in the VGluT3-Cre mice (one-way ANOVA; *N*_control _= 8; *N*_excitatory _= 13; *N*_inhibitory _= 15). ***C***, Schematic representation of SIT protocol: for 10 min two experimental animals from the same treatment group (e.g., KO vs KO, excitatory vs excitatory) was placed into a neutral, anxiogenic environment to freely interact. Under the figure Gantt diagrams visually represent the behavior of all experimental animals during SIT. Different behaviors are represented by different colors. ***D***, Schematic representation of RIT protocol: for 10 min an unfamiliar, physically smaller, but adult conspecific was placed into the home cage of the experimental mouse to freely interact. Under the figure Gantt diagrams visually represent the behavior of all experimental animals during RIT. Different behaviors are represented by different colors. ***E***, Frequency of aggressive behavior did not differ between WT and VGluT3 KO mice during RIT (*t* test; *N*_WT _= 10; *N*_KO _= 11). ***F***, Frequency of friendly social behavior was decreased in the inhibitory group of VGluT3-Cre mice in RIT (one-way ANOVA; *N*_control _= 8; *N*_excitatory _= 13; *N*_inhibitory _= 14). Data are expressed as average ± SEM. Empty squares represent individual values. For more details see Extended Data Tables to Figure 3. KO, knock-out; RIT, resident intruder test; SIT, social interaction test; VGluT3, vesicular glutamate transporter type 3; WT, wild-type. ^##^*p* < 0.01 versus mouse; **p < 0.01 versus WT; ^==^*p* < 0.01 versus control; ^@^*p* < 0.05 versus excitatory; ^–^*p* < 0.01 versus inhibitory.

The test was conducted in a transparent Plexiglass chamber (35 cm × 20 cm × 25 cm, Geo Maxi mini aquarium) with bedding (Fig. 3*C*). On previous day mice were put into the respective chamber for 2 × 15 min, 4 h apart, for habituation. During the experiment, two animals from the same group (e.g., KO vs KO), roughly weighting the same, were put into the chamber. The mice could freely behave for 10 min under normal lighting.

Social (e.g., sniffing), aggressive (e.g., biting, aggressive dominance), and defensive behaviors were analyzed for both test animals. Any other type of behavior was labeled as “other.”

#### Resident intruder

An unfamiliar, physically smaller, but adult conspecific was put into the homecage of the test animals. For 10 min, the animals could freely behave (Fig. 3*D*). Only the behavior of the test animal was analyzed (with similar parameters as in case of SIT).

#### EPM

The animals were placed onto the middle, central zone of the EPM apparatus (height, 25 cm; width, 7 cm; length, 30 cm; 67 cm above the floor) with two open and two closed arms aimed to measure anxiety (Fig. 2*D*). The mice could behave freely for 5 min in bright light (120 lux).

Open arm entries reflecting mobility independent anxiety were calculated:

$$\frac{\mathrm{Open}}{\mathrm{Total}}(\text{\%})=\frac{\mathrm{Number}\;\mathrm{of}\;\mathrm{open}\;\mathrm{arm}\;\mathrm{entries}}{\mathrm{Sum}\;\mathrm{of}\;\mathrm{open}+\mathrm{closed}\;\mathrm{arm}\;\mathrm{entries}}.$$

The frequency of risk assessment (RA) behavior (head dipping, stretched attend posture, and rearing) and grooming were also evaluated.

#### Y-maze

**Figure 4.**
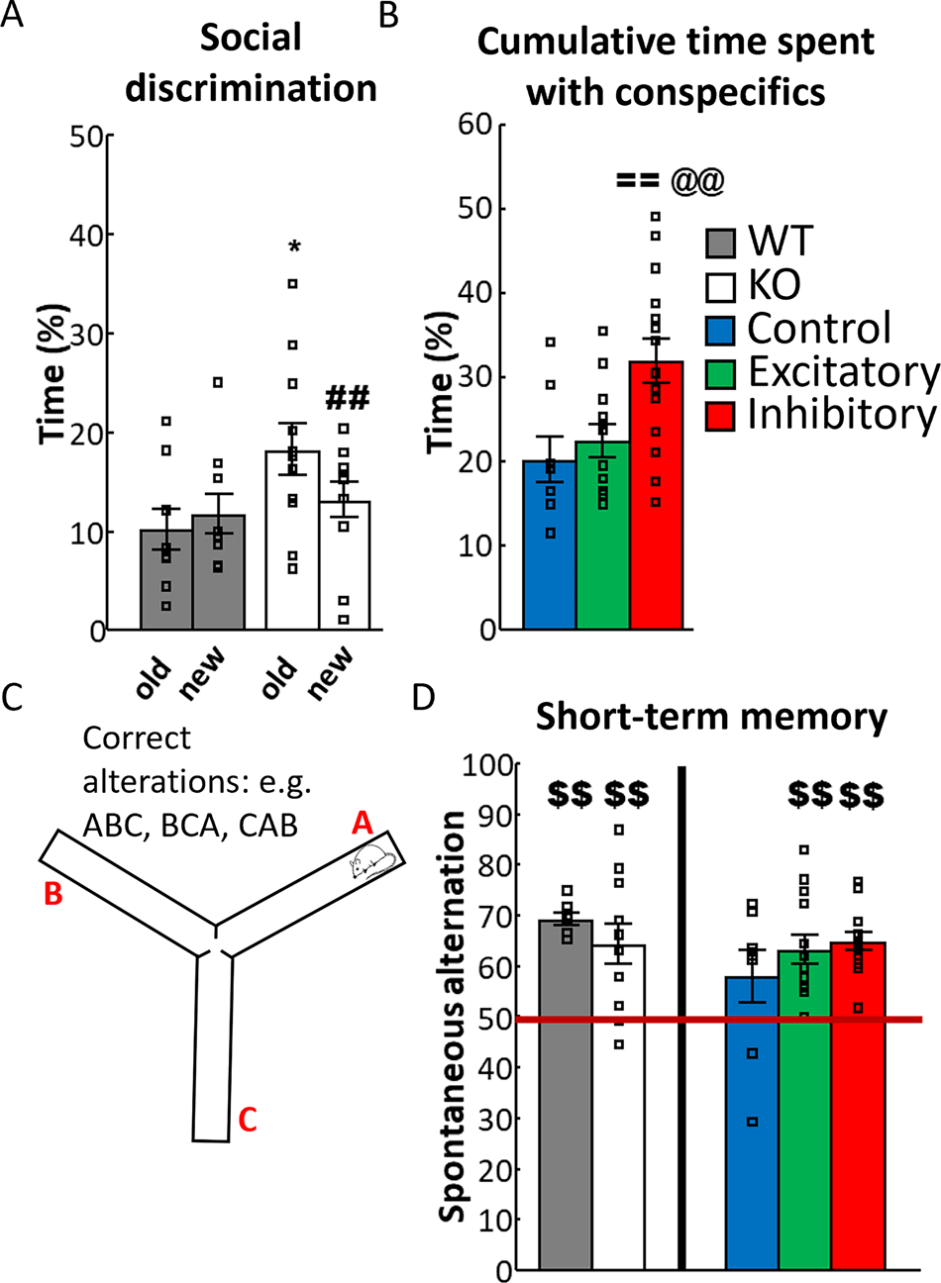
Results of social and working memory experiments. ***A***, SDT conducted 1 d after sociability test (Fig. 2*A*) showed a preference for the familiar, “old” mouse in VGluT3 KO mice (repeated-measures ANOVA; *N*_WT _= 10; *N*_KO _= 11). ***B***, In SDT for VGluT3-Cre mice, there were no differences between the groups regarding their social memory (repeated-measures ANOVA), but the cumulative time spent with social stimuli showed an increased social interest in the inhibitory group (one-way ANOVA; *N*_control _= 8; *N*_excitatory _= 12; *N*_inhibitory _= 15). ***C***, Schematic figure of Y-maze test. Three identical arms placed perpendicular can be freely explored by the experimental animal for 5 min. Proper working memory is reflected by consecutive, different arm entries, called spontaneous alternation. ***D***, All groups had intact working memory compared with random 50% except for the VGluT3-Cre control group (single sample *t* test; *N*_WT _= 10; *N*_KO _= 11; *N*_control _= 8; *N*_excitatory _= 13; *N*_inhibitory _= 14). Data are expressed as average ± SEM. Empty squares represent individual values. For more details see Extended Data Tables to Figure 4. KO, knock-out; SDT, social discrimination test; VGluT3, vesicular glutamate transporter type 3; WT, wild-type. ^##^*p* < 0.01 versus “old” mouse; ^$$^*p* < 0.01 versus random 50; ^==^*p* < 0.01 versus control; ^@@^*p* < 0.01 versus excitatory.

This test detects the short-term learning and memory of rodents, reflected by continuous spontaneous alternation (Hughes, 2004). The apparatus consists of three arms (A, B, and C; 25 cm × 5 cm × 21 cm) at 120°, connected by a central zone (Fig. 4*C*). No extra external spatial cues were used during the experiment. The consecutive arm entries reflect intact short-term memory. Mice were placed at the end of arm A and were allowed to explore the maze freely for 5 min.

Spontaneous alternation was calculated as follows:

$$\mathrm{Spontaneous}\;\;\mathrm{alternation}=\frac{“\mathrm{correct}”\;\mathrm{alternation}}{\mathrm{sum}\;\mathrm{of}\;\mathrm{all}\;\mathrm{arm}\;\mathrm{entries}-2}\;\times \;100,$$

where “correct” alternation is the entry into all three arms on consecutive choices (i.e., ABC, but not CAC; Fazekas et al., 2019; Chaves et al., 2022).

### Immunohistochemistry and microscopy

In VGluT3-Cre mice, virus infection and expression had to be validated. Nickel-3.3′-diaminobenzidine (Ni-DAB, Sigma-Aldrich) staining was conducted on midbrain slices containing MRR (Biro et al., 2017; Chaves et al., 2022). The slices were incubated in anti-RFP primer solution (1:4,000, rabbit, #600-401-379, Rockland) for two nights on 4°C. The secondary solution consisted of biotinylated (biotin-SP) anti-rabbit antibody (1:1,000 donkey, #711-065-152, Jackson). The signal was strengthened by avidin–biotin complex (1:1,000, ABC; Vector Laboratories) and then developed in the presence of DAB (10 mg/ml) and 1% NiNH_4_SO_4_ (Sigma-Aldrich) and H_2_O_2_ (Sigma-Aldrich). The mounting was done in gelatine (G9391, Sigma-Aldrich), and then the slides were dehydrated in xylol and covered with DPX (Sigma-Aldrich).

The Ni-DAB–stained slices were evaluated with Olympus DP70 light microscope (4× magnification). The virus expression was examined between −4.04 and −4.96 mm from bregma. In case of no or unilateral staining, or infected other brain regions (e.g., dorsal raphe), the test animal and associated data were excluded from the statistical analysis (17/53; Fig. 1*B*).

To verify whether only VGluT3+ cells in the MRR expressed the virus, double immunofluorescent staining was conducted (Chaves et al., 2022). For two nights, slides were incubated in anti-RFP (1:1,000, rat, #5f8-100, ChromoTek) and anti-VGluT3 (1:600, rabbit, #135203, Synaptic Systems) primer solutions on 4°C, then in secondary anti-rabbit antibody conjugated with Alexa-488 (1:500, goat, #111-545-003, Jackson), and anti-rat antibody conjugated with Alexa-594 (1:1,000, goat, #A11029, Invitrogen). Slices were mounted with gelatine and covered with Mowiol (Sigma-Aldrich). The double immunofluorescent staining was evaluated by C2 confocal laser-scanning microscope (Nikon Europe; 20× magnification; Fig. 1*C*).

c-Fos cell counting in brain sites relevant to social behavior and well-known projection areas of MRR (prelimbic cortex, infralimbic cortex and anterior cingulate, medial septum, CA1, CA2, and dentate gyrus) as well as in MRR was also done by Ni-DAB staining. Slices were incubated in the anti-c-Fos primer solution (1:1,000, rabit, #sc-52, Santa Cruz) for two nights on 4°C. The rest of the protocol was the same as described above. The stained slices were scanned by Pannoramic MIDI II Slide scanner (3DHISTECH) and cell bodies (three slides/animal) were counted by ImageJ.

### Human brain samples

**Figure 5.**
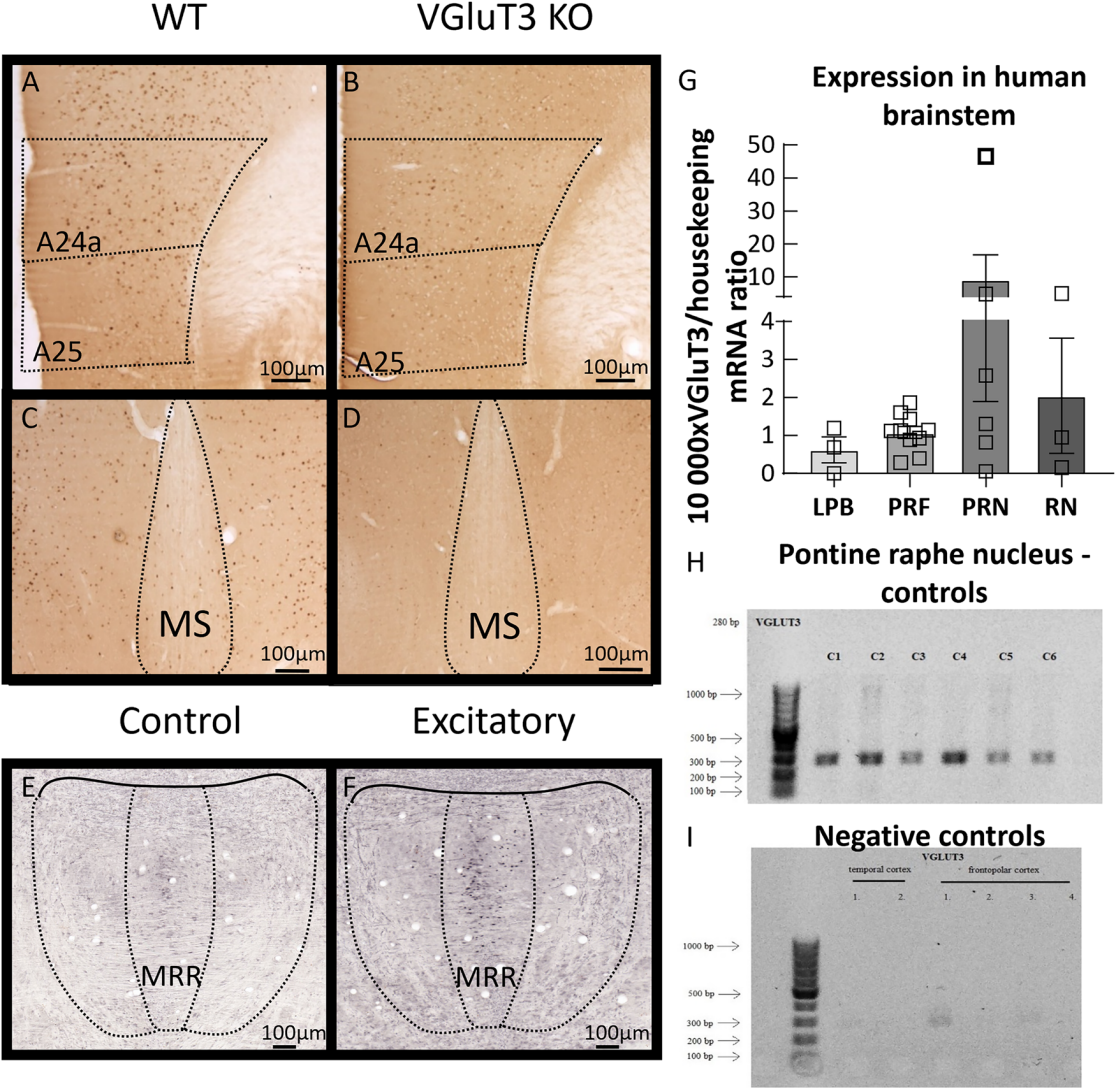
c-Fos staining in the mouse brain and VGluT3 mRNA expression in healthy human samples. ***A–D***, There are decreased numbers of c-Fos-positive cells in the KO (***B***,***D***) compared with WT (***A***,***C***) mice in the mPFC (***A***,***B***) and MS (***C***,***D***) after SIT (*N*_WT _= 5; *N*_KO _= 8). ***E***,***F***, Functional DREADD excitation is reflected by increased c-Fos activity in the MRR of VGluT3-Cre mice (*N*_control _= 6; *N*_excitatory _= 3). Scale bar, 100 μm. ***G***, Relative expression of VGluT3 mRNA to the measured housekeeping gene (GAPDH) in the investigated brainstem areas (LPB: *N* = 3; PRF: *N* = 10; PRN: *N* = 6; RN: *N* = 3). ***H***, After RT-qPCR, samples containing VGluT3 cDNA were run with gel electrophoresis and sequencing (BIOMI) to validate the specificity of primers. Brain samples from pontine raphe nuclei, which contain the human equivalent of mouse MRR, all expressed VGluT3 (*N* = 6). ***I***, Different cortical areas of healthy human samples were used as negative controls (*N* = 6). Data are expressed as average ± SEM. Empty squares represent individual values. For more details see Extended Data Tables to Figure 5. bp, base pair; DREADD, designer receptors exclusively activated by designer drugs; KO, knock-out; LPB, lateral parabrachial nucleus; mPFC, medial prefrontal cortex; MRR, median raphe region; MS, medial septum; PRF, pontine reticular formation; PRN, pontine raphe nucleus; RN, midbrain raphe nuclei; RT-qPCR, reverse transcription quantitative polymerase chain reaction; SIT, social interaction test; VGluT3, vesicular glutamate transporter type 3; WT, wild-type.

The study was approved by the Hungarian Medical Research Council – Scientific and Research Ethical Committee (#40197-2/2019/EKU), in accordance with the Ethical Rules for Using Human Tissues for Medical Research in Hungary (HM 34/1999) and the Code of Ethics of the World Medical Association (Declaration of Helsinki). Postmortem brain samples were obtained from the Human Brain Tissue Bank, Semmelweis University. The brains of 11 individuals were used in the study. Average ± SEM age was 56.4 ± 54.33 years, while the postmortem delay was 5.72 ± 0.71-h (for details see Extended Data Table to Fig. 5-3).

Total RNA was isolated by RNeasy Mini Kit (Biomarker) according to the manufacturer's instructions. RNA was diluted into RNase-free water. The quality and quantity of extracted RNA was determined using NanoDrop ND-1000 Spectrophotometer (Thermo Fisher Scientific), and only those with A260/A280 ratio between 1.8 and 2.1 were used in subsequent experiments. The isolated RNA concentration was calculated and normalized with RNase-free water and reverse transcribed into cDNA using SuperScript II reverse transcriptase kit (Invitrogen). After 10-fold dilution, 2.5 μl of the resulting cDNA was used as template in PCR reactions using SYBR Green dye (Sigma). The PCR reactions were performed on CFX-96 C1000 Touch Real-Time System (Bio-Rad Laboratories) with iTaq DNA polymerase (Bio-Rad Laboratories) in total volumes of 12.5 μl under the following conditions: 95°C for 3 min, followed by 35 cycles of 95°C for 0.5 min, 60°C for 0.5 min, and 72°C for 1 min. A melting curve was performed at the end of amplification cycles to verify the specificity of the PCR products. All the measurements were conducted in duplicates. The primers used for RT-qPCR were synthesized by Integrated DNA Technologies and used at 300 nM final concentration. Sequences of intron spanning primers were designed based on the common parts of the two splice variants (NM_001145288.2 and NM_139319.3, 122nt-3834nt) to eliminate detection of genomic DNA. The forward primer was GTCTGTCCCCTCATTGTCGG, while the reversed primer was CACAATTCTGGGAGGTGGCT, with an expected product size of 280 bp. Final cDNA products were sequenced by BIOMI and showed matching to the originally used primers (available upon request). One-third of the amplified PCR product was loaded in agarose gel (1.2%) containing Eco Safe nucleic acid staining solution (1:20,000, Pacific Image Electronics) and the electrophoresis was conducted in 1× TAE (40 mM Tris-acetate, 1 mM EDTA, pH 8.0) buffer. The applied separation voltage was 100 V. After the electrophoresis, the DNA bands were visualized by UV transillumination.

### Statistical analysis

StatSoft 13.4 software was used. Outliers were defined as data points outside the interval of group mean ± 2× standard deviation for the given parameter and thus, were excluded from the analysis. This outlier analysis revealed uncharacteristic behavior resulted in the exclusion of the following number of animals: one WT mouse in the OF test; three WT mice in the Y-maze test; and one WT and one KO mice in sociability and SDTs. Similarly, in the case of VGluT3-Cre mice, the following number of animals were excluded: one mouse in the inhibitory group in the Y-maze test; two mice in the excitatory group during sociability test—object habituation phase, out of which one had to be also excluded from the rest of the experiment (sociability phase, SDT) as well. In the SIT and the EPM test, 1-1 mouse from the excitatory group was excluded. Finally, two mice (one control and one inhibitory) had to be removed from the RIT analysis.

To compare WT and KO animals, we used a *t* test (effect of genotype), while for the behavior of VGluT3-Cre mice, one-way analysis of variance (ANOVA; effect of manipulation) was used. The c-Fos-positive cell counting was done for every brain region separately, as staining was conducted also separately. Therefore, the groups were also compared with *t* test (WT-KO, effect of genotype; VGluT3-Cre control-excitatory, effect of manipulation). However, post hoc correction for multiple comparisons was also done by Benjamini–Hochberg correction (FDR, false discovery rate). SI and spontaneous alteration were tested with single-sample *t* test against 50%, while SD values were tested against 0%. The comparison of sides (left vs right), social preferences in sociability (mouse vs cage), and social discrimination (old vs new) were conducted by mixed-design ANOVA (effect of genotype or manipulation, effect of choice as repeated measure). For aggressive behavior of WT-KO animals in SIT, contingency table was made and Pearson’s chi-square test was conducted. Fisher LSD was used for post hoc analysis. Data were expressed as mean ± SEM and *p* < 0.05 was considered statistically significant, while 0.1 < *p* < 0.05 was accepted as a marginal difference.

## Results

### VGluT3 KO studies

#### Mobility

There was no difference between the WT and VGluT3 KO animals in the distance moved in OF (Fig. 2*B*; Extended Data Table to Fig. 2-1), frequency of closed arm entries on EPM (Extended Data Table to Fig. 2-2), or arm entries in Y-maze (Extended Data Table to Fig. 4-2).

10.1523/ENEURO.0332-23.2024.f2-1 Figure 2-1 **Results of open field test - VGluT3 WT-KO animals.** Degree of freedom (df) for the two-sample t-tests was 18. Data are expressed as mean ± SEM. WT: wild-type; KO: knock-out. ** p < 0.01 vs WT. Download Figure 2-1, DOCX file.

10.1523/ENEURO.0332-23.2024.f2-2 Figure 2-2 **Results of elevated plus-maze test - VGluT3 WT-KO animals. WT-KO:** Degree of freedom (df) for the two-sample t-test (all parameters) is 19. Marginal effects are in brackets (). Data are expressed in mean ± SEM. WT: wild-type; KO: knock-out; RA: risk assessment; SAP: stretched attend posture. * p < 0.05 vs WT. Download Figure 2-2, DOCX file.

10.1523/ENEURO.0332-23.2024.f2-3 Figure 2-3 **Results of open field test - VGluT3-Cre animals.** Degree of freedom (df) for the one-way ANOVA was (2,32). Marginal effects are in brackets (). Data are expressed as mean ± SEM. Download Figure 2-3, DOCX file.

10.1523/ENEURO.0332-23.2024.f2-4 Figure 2-4 **Results of elevated plus-maze test – VGluT3-Cre animals.** Degree of freedom (df) for the one-way ANOVA for the frequency and time (%) spent in different zones is (2,32), while for the risk assessment behaviour is (2,31). Marginal effects are in brackets (). Data are expressed in mean ± SEM. RA: risk assessment; SAP: stretched attend posture. == p < 0.01 vs control; & p < 0.05 vs inhibitory Download Figure 2-4, DOCX file.

#### Anxiety-like behavior

In the OF, the frequencies of entering different zones did not show any differences. However, the KO animals spent less time in the centrum than the WT (*t*_(18) _= 2.963; *p* = 0.008; *t* test; Fig. 2*C*; Extended Data Table to Fig. 2-1). This increased anxiety-like behavior was confirmed on the EPM: KO animals tended to enter the open arm less than the WT (open/total%: *t*_(19) _= 2.019; *p* = 0.058; *t* test; Fig. 2*E*; Extended Data Table to Figure 2-2). Additionally, the frequency of rearing (risk assessment) was lower for the KO animals (*t*_(19) _= 2.645; *p* = 0.016; *t* test). Overall, the KO group exhibited less risk assessment behavior than WT (RA_WT _= 67.500 ± 5.584; RA_KO _= 49.091 ± 6.511; *t*_(19) _= 2.125; *p* = 0.047).

#### Social interest

During object habituation, KO spent more cumulative time with the two objects than the WT (*F*_(1,17) _= 4.378; *p* = 0.052) without side preferences. This is also reflected by the reduced time spent with “other” behavior in KO mice (*t*_(17) _= 2.09; *p* = 0.052; Extended Data Table to Figure 3-1), during which they did not investigate the two empty cages.

During the sociability phase, a significant difference was detected in sniffing frequencies for the effect of choice (*F*_(1,18) _= 16.503; *p* < 0.001) and a tendency for genotype × choice interaction (*F*_(1,18) _= 3.327; *p* = 0.085; Extended Data Table to Fig. 3-2), indicating preference for a social stimulus. For the percentage of time, there was a significant effect of genotype (*F*_(1,18) _= 6.066; *p* = 0.024), choice (*F*_(1,18) _= 47.541; *p* < 0.001), and genotype × choice interaction (*F*_(1,18) _= 5.246; *p* = 0.034), supporting the frequency data. Post hoc analysis confirmed social preference in both genotypes (WT: *p* = 0.006; KO: *p* < 0.001). Moreover, KO mice spent more time with the conspecific than the WT (*p* = 0.002; Fig. 3*A*). Additionally, KO group spent less time with “other” behavior (*t*_(18) _= 2.463; *p* = 0.024), during which they did not investigate the two stimulus cages. Both groups had adequate—over random chance 50%—SI (WT: *t*_(8) _= 3.672, *p* = 0.006; KO: *t*_(10) _= 10.630, *p* < 0.001) with significantly higher value in KO animals (*t*_(18) _= −2.087; *p* = 0.051). These results indicate an increased interest toward conspecifics in the VGluT3 KO mice.

10.1523/ENEURO.0332-23.2024.f3-1 Figure 3-1 **Results of sociability test – object habituation phase – VGluT3 WT-KO animals.** Degree of freedom (df) for the two-sample t-test (frequency and time [%] of ‘other’ behaviour) was 17. Degree of freedom in the repeated-measures ANOVA (frequency and time [%] of left vs right cage) was (1,17) for all effects. Data are expressed in mean ± SEM. WT: wild-type; KO: knock-out. * p < 0.05 vs WT. Download Figure 3-1, DOCX file.

10.1523/ENEURO.0332-23.2024.f3-2 Figure 3-2 **Results of sociability test -sociability phase – VGluT3 WT-KO animals.** Degree of freedom (df) for the two-sample t-test (frequency and time [%] of ‘other’ behaviour; SI) is 18. Degree of freedom in the repeated-measures ANOVA (frequency and time [%] of left vs right cage) is (1,18) for all effects. Marginal effects are in brackets (). Data are expressed in mean ± SEM. WT: wild-type; KO: knock-out; SI: sociability index. ## p < 0.01 vs cage; ** p < 0.01, * p < 0.05 vs WT; $$ p < 0.01 vs random 50. Download Figure 3-2, DOCX file.

#### Social behavior

During SIT frequency of defensive and “other” behavior showed only marginal genotype effect (*t*_(19) _= −1.808; *p* = 0.086 and *t*_(19) _= −1.936; *p* = 0.068, respectively; Extended Data Table to Fig. 3-3). However, there was an increase in the percentage of time (*t*_(19) _= −2.182; *p* = 0.042) and frequency of aggressive behavior (*t*_(19) _= −2.762; *p* = 0.012) in KO compared with WT. Since the values were low compared with the full length of the test (10 min), our continuous data were transformed into discrete variables: those animals who showed aggression were marked with “yes,” while those who did not were marked with “no” and a contingency table was created. Pearson’s chi-square test showed a significant difference (*χ*^2^ = 7.636; *p* = 0.006): 6 KO animals out of 11 exhibited aggressive behavior, while the WT group showed none (Fig. 3*B*,*C*; Extended Data Table to Fig. 3-4).

10.1523/ENEURO.0332-23.2024.f3-3 Figure 3-3 **Results of social interaction test – VGluT3 WT-KO animals.** Degree of freedom (df) for the two-sample t-test (all parameters) is 19. Marginal effects are in brackets (). Data are expressed in mean ± SEM. WT: wild-type; KO: knock-out. * p < 0.05 vs WT. Download Figure 3-3, DOCX file.

10.1523/ENEURO.0332-23.2024.f3-4 Figure 3-4 **VGluT3 WT-KO contingency table**. The table shows the numbers of animals who exhibited aggressive behaviour in social interaction test, followed by a Pearson Chi-square test. WT: wild-type; KO: knock-out. Download Figure 3-4, DOCX file.

In RIT, there was no difference between groups, and only a marginal effect of genotype was found in the frequency of defensive behavior (*t*_(19) _= 1.878; *p* = 0.076; (Fig. 3*D*,*E*; Extended Data Table to Fig. 3-5). These results show a context-dependent role of VGluT3 in aggressive behavior.

10.1523/ENEURO.0332-23.2024.f3-5 Figure 3-5 **Results of resident intruder test – VGluT3 WT-KO animals.** Degree of freedom (df) for the two-sample t-test (all parameters) is 19. Marginal effects are in brackets (). Data are expressed in mean ± SEM. WT: wild-type; KO: knock-out. == p < 0.01 vs control. Download Figure 3-5, DOCX file.

10.1523/ENEURO.0332-23.2024.f3-6 Figure 3-6 **Results of sociability test – object habituation phase – VGluT3-Cre animals.** Degree of freedom (df) for the one-way ANOVA (frequency and time [%] of ‘other’ behaviour) was (2,31). Degree of freedom in the repeated-measures ANOVA (frequency and time [%] of left vs right) is (2,31) for the effect of manipulation and manipulation × choice interaction, while (1,32) for the effect of choice. Marginal effects are in brackets (). Data are expressed in mean ± SEM. Download Figure 3-6, DOCX file.

10.1523/ENEURO.0332-23.2024.f3-7 Figure 3-7 **Results of sociability test – sociability phase – VGluT3-Cre animals.** Degree of freedom (df) for the one-way ANOVA (frequency and time [%] of ‘other’ behaviour) is (2,32). Degree of freedom in the repeated-measures ANOVA (frequency and time [%] of mouse vs cage) is (2,32) for the effect of manipulation and manipulation × choice interaction, while (1,32) for the effect of choice. Data are expressed in mean ± SEM. SI: sociability index. ## p < 0.01 vs cage; - p < 0.05 vs inhibitory; $ p < 0.05 vs random 50. Download Figure 3-7, DOCX file.

10.1523/ENEURO.0332-23.2024.f3-8 Figure 3-8 **Results of social interaction test – VGlut3-Cre animals.** Degree of freedom (df) for the one-way ANOVA (all parameters) is (2,32). Marginal effects are in brackets (). Data are expressed in mean ± SEM. = p < 0.05 vs control; @ p < 0.05 vs excitatory Download Figure 3-8, DOCX file.

10.1523/ENEURO.0332-23.2024.f3-9 Figure 3-9 **Results of resident intruder test – VGluT3-Cre animals. VGluT3-Cre:** Degree of freedom (df) for the one-way ANOVA (all parameters) is (2,31). Data are expressed in mean ± SEM. WT: wild-type; KO: knock-out. == p < 0.01 vs control; @ p < 0.05 vs excitatory. Download Figure 3-9, DOCX file.

#### Social discrimination

There was a marginal effect of genotype in the percentage of time spent with stimulus animals (*F*_(1,18) _= 3.142; *p* = 0.093) and a significant genotype × choice interaction (*F*_(1,18) _= 4.281; *p* = 0.053). Post hoc analysis of the interaction showed that KO mice spent more time with the already familiar, “old” mouse compared with the WT group (*p* = 0.015) and also compared with the unknown, “new” mouse (*p* = 0.031; Fig. 4*A*; Extended Data Table to Fig. 4-1). This was also reflected by SD: WT animals did not differ significantly from random chance 0, unlike the KO group (*t*_(10) _= −2.352; *p* = 0.040). Thus, the SD difference between the groups was significant (*t*_(17) _= 2.503; *p* = 0.023; Extended Data Table to Fig. 4-1). Nevertheless, their average SD was −19.329 ± 8.217, which reflects a preference for the “old” mouse.

10.1523/ENEURO.0332-23.2024.f4-1 Figure 4-1 **Results of social discrimination test – VGluT3 WT-KO animals.** Degree of freedom (df) for the two-sample t-test (frequency and time [%] of ‘other’ behaviour; SI) is 18. Degree of freedom in the repeated-measures ANOVA (frequency and time [%] of ‘old’ vs ‘right’ mouse) is (1,18) for all effects. Marginal effects are in brackets (). Data are expressed in mean ± SEM. WT: wild-type; KO: knock-out; SD: social discrimination index. * p < 0.05 vs WT; # p < 0.05 vs cage; $ p < 0.05 vs random 0. Download Figure 4-1, DOCX file.

10.1523/ENEURO.0332-23.2024.f4-2 Figure 4-2 **Results of Y-maze test – VGluT3 WT-KO animals.** Degree of freedom (df) for the two-sample t-test for locomotion is 19, for alteration is 16. Data are expressed in mean ± SEM. WT: wild-type; KO: knock-out. $$ p < 0.01 vs random 50. Download Figure 4-2, DOCX file.

10.1523/ENEURO.0332-23.2024.f4-3 Figure 4-3 **Results of social discrimination test – VGluT3-Cre animals.** Degree of freedom (df) for the one-way ANOVA (frequency and time [%] of ‘other’ behaviour) is (2,32). Degree of freedom in the repeated-measures ANOVA (frequency and time [%] of mouse vs cage) is (2,32) for the effect of manipulation and manipulation × choice interaction, while (1,32) for the effect of choice. Marginal effects are in brackets (). Data are expressed in mean ± SEM. SD: social discrimination index. # p < 0.05 vs cage; == p < 0.01 vs control; @@ p < 0.01 vs excitatory. $ p < 0.05 vs random 0. Download Figure 4-3, DOCX file.

10.1523/ENEURO.0332-23.2024.f4-4 Figure 4-4 **Results of Y-maze test – VGluT3-Cre animals.** Degree of freedom (df) for the one-way ANOVA for locomotion is (2,32), while for alteration is (2,31). Data are expressed in mean ± SEM. $$ p < 0.01 vs random 50. Download Figure 4-4, DOCX file.

#### Working memory

In the Y-maze test, both groups showed higher than random chance spontaneous alteration (WT: *t*_(6) _= 15.154, *p* < 0.001; KO: *t*_(10) _= 3.608, *p* = 0.005), indicating intact working memory, without genotype differences (Fig. 4*D*; Extended Data Table to Fig. 4-2).

#### Neuronal activity

Ninety minutes after, SIT neuronal activation was measured by c-Fos immunohistochemistry. There was a significant decrease in the number of c-Fos-positive cells in the medial septum (*t*_(11) _=_ _4.199; *p* = 0.001) and infralimbic cortex (*t*_(11) _= 3.402; *p* = 0.006) of VGluT3 KO mice compared with WT. Other brain areas did not show significant difference after FDR correction (Fig. 5*A–D*; Extended Data Table to Fig. 5-1).

10.1523/ENEURO.0332-23.2024.f5-1 Figure 5-1 **Results of c-Fos positive cell counting – VGluT3 WT-KO animals.** VGluT3 KO mice showed decreased activity after social test in their anterior cingulate, infralimbic cortex and medial septum. Degree of freedom (df) for the two-sample t-test is 11. Benjamini-Hochberg (FDR, false discovery rate) post-hoc correction was used, thus a p-value was considered significant from 0.016. Data are expressed in mean ± SEM. WT: wild-type; KO: knock-out. * p < 0.05, ** p < 0.01 vs WT Download Figure 5-1, DOCX file.

10.1523/ENEURO.0332-23.2024.f5-2 Figure 5-2 **Results of c-Fos positive cell counting – VGluT3-Cre animals.** The excitatory group showed increased neuronal activity only in the viral injected target area, the median raphe region. Degree of freedom (df) for the two-sample t-test is 7. Benjamini-Hochberg (FDR, false discovery rate) post-hoc correction was used, thus a p-value was considered significant from 0.008. Data are expressed in mean ± SEM. ## p < 0.01 vs Control. Download Figure 5-2, DOCX file.

10.1523/ENEURO.0332-23.2024.f5-3 Figure 5-3 **Subject information for the RT-PCR measurements in the brainstem of humans.** Download Figure 5-3, DOCX file.

10.1523/ENEURO.0332-23.2024.f5-4 Figure 5-4 **RT-PCR results of human samples.** Four major brainstem nuclei were investigated, out of which all showed VGluT3 expression on an mRNA level. The results show averaged and normalized CT values. VGluT3: vesicular glutamate transporter type 3. Download Figure 5-4, DOCX file.

### MRR-VGluT3 studies

#### Mobility

MRR-VGluT3 manipulation only tended to affect the distance traveled in OF (*F*_(2,32) _= 3.04; *p* = 0.062; Fig. 2*B*; Extended Data Table to Fig. 2-3). Post hoc analysis showed that the inhibitory group moved less compared with control (*p* = 0.082) and excitatory groups (*p* = 0.029). Moreover, on EPM (Extended Data Table to Fig. 2-4) and in Y-maze (Extended Data Table to Fig. 4-4), the locomotion did not differ between the groups.

#### Anxiety-like behavior

In the OF, neither the frequencies nor the percentage of time spent in the centrum differed between the groups (Fig. 2*C*; Extended Data Table to Fig. 2-3). On the other hand, on the EPM MRR-VGluT3 manipulation significantly influenced the open/total% values (*F*_(2,32) _= 3.828; *p* = 0.032). According to the post hoc analysis, the excitatory group entered the open arms more often than control group (*p* = 0.009; Fig. 2*E*; Extended Data Table to Fig. 2-4). The frequency of rearing showed a tendency (*F*_(2,31) _= 2.769; *p* = 0.078), while head dipping was significantly influenced by MRR-VGluT3 manipulation (*F*_(2,31) _= 3.572; *p* = 0.040). Post hoc test of head dipping revealed an increase in the excitatory group compared with both control and inhibitory groups (*p* < 0.05). Based on this, the excitation of MRR-VGluT3 neurons was proved to be anxiolytic.

#### Social interest

During object habituation, there was a marginal effect of choice (*F*_(1,32) _= 3.990; *p* = 0.055), without significant post hoc effects between the two empty cages (Extended Data Table to Fig. 3-6), and thus, we did not apply correction for side preference during subsequent analysis.

During sociability phase, frequencies only showed significant effect of choice (*F*_(1,32) _= 33.030; *p* < 0.001), indicating that all animals preferred the stimulus animal, regardless of MRR-VGluT3 manipulation (Extended Data Table to Fig. 3-7). However, percentage of time spent with the stimulus mouse was significantly influenced by MRR-VGluT3 manipulation (*F*_(2,32) _= 6.002; *p* < 0.006) and choice (*F*_(1,32) _= 88.767; *p* < 0.001). Post hoc analysis revealed that all mice spent more time with the conspecific (*p* < 0.001). Moreover, the excitatory group spent less time with the two cages together compared with inhibitory group (*p* < 0.001; Fig. 3*A*). Additionally, there was a significant effect of MRR-VGluT3 manipulation for percentage of time spent with “other” behavior (*F*_(2,32) _= 5.580; *p* = 0.008), during which the test mice did not investigate the two stimulus cages. More precisely, the excitatory group spent more time with “other” behavior than the inhibitory group (*p* = 0.002). All three groups had adequate, higher than 50% SI (control: *t*_(7) _= 13.670, *p* = 0.001; excitatory: *t*_(11) _= 7.324, *p* < 0.001; inhibitory: *t*_(14) _= 6.658, *p* < 0.001), indicating preference for social stimulus, without differences between the groups (Extended Data Table to Fig. 3-7).

#### Social behavior

In SIT, there was a marginal effect of MRR-VGluT3 manipulation in the frequency of social behavior (*F*_(2,32) _= 2.702; *p* = 0.082; Extended Data Table to Fig. 3-8). The frequency of “other,” nonsocial behavior had a significant effect of MRR-VGluT3 manipulation (*F*_(2,32) _= 4.089; *p* = 0.026), with higher levels in the inhibitory than in control (*p* = 0.030) or excitatory groups (*p* = 0.018). Additionally, MRR-VGluT3 manipulation tended to influence the percentage of time spent with “other,” nonsocial behavior as well (*F*_(2,32) _= 2.613; *p* = 0.089; Extended Data Table to Fig. 3-8).

During RIT, MRR-VGluT3 manipulation significantly influenced social behavior (*F*_(2,31) _= 5.938; *p* = 0.006). Post hoc test revealed that the inhibitory group initiated fewer social contacts compared with control (*p* = 0.003) and excitatory (*p* = 0.024) groups. The frequency of “other,” nonsocial behavior was also significant influenced (*F*_(2,31) _= 5.308; *p* = 0.010); the inhibitory group had lower frequency compared with control (*p* = 0.005) and excitatory (*p* = 0.025) groups (Fig. 3*D*,*F*; Extended Data Table to Fig. 3-9).

#### Social discrimination

None of the groups could differentiate between the stimulus animals (Extended Data Table to Fig. 4-3). However, there was a significant effect of MRR-VGluT3 manipulation in both the frequencies of interaction (*F*_(2,32) _= 3.336; *p* = 0.048) and percentage of time spend with it (*F*_(2,32) _= 7.070; *p* = 0.003). Post hoc test showed that inhibitory group had greater interaction frequencies compared with control (*p* = 0.020), which differed marginally from the excitatory groups (*p* = 0.092; Extended Data Table to Fig. 4-3). In the meantime, post hoc test revealed that the inhibitory group spent more time with the two conspecifics together compared with both control (*p* = 0.003) and excitatory groups (*p* = 0.005; Fig. 4*B*; Extended Data Table to Fig. 4-3). Accordingly, the frequency (*F*_(2.32) _= 2.586; *p* = 0.091) and percentage of time (*F*_(2,32) _= 6.526; *p* = 0.004) spent with “other,” nonsocial behavior showed marginal effect of MRR-VGluT3 manipulation. Post hoc analysis confirmed that the inhibitory group initiated less often (frequency) and spent less time with nonsocial behavior compared with control (*p* = 0.004) and excitatory (*p* = 0.007) groups. Interestingly, this increase in social interest (reflected by the higher time spent with both stimulus animals) was observable 24 h after MRR-VGluT3 inhibition was induced during sociability test.

#### Working memory

Spontaneous alteration was intact for the MRR-VGluT3 manipulated groups (excitatory: *t*_(12) _= 4.795, *p* < 0.001; inhibitory: *t*_(12) _= 8.475, *p* < 0.001; single-sample *t* test against 50), but not for control group (control: *t*_(7) _= 1.605, *p* = 0.153; Fig. 4*D*). However, in direct comparison, there were no significant differences between the groups (Extended Data Table to Fig. 4-4).

#### Neuronal activity

Only the MRR region showed statistically significant neuronal activation. As expected, after CNO injection, we could detect increased number of c-Fos-positive cells in the stimulatory DREADD group compared with control virus injected one (*t*_(7) _= −14.987; *p* < 0.001; Fig. 5*E*,*F*), confirming that the excitatory DREADD receptors were functional and induced neuronal activation. However, its projection areas did not show increased neuronal activity (Extended Data Table to Fig. 5-2).

### Human brain PCR results

VGluT3 mRNA levels were investigated in four human brainstem areas: lateral parabrachial nucleus, pontine reticular formation, pontine raphe nucleus, and midbrain raphe nuclei (Fig. 5*G*). As negative control, temporal and frontopolar cortex samples were used (Fig. 5*H*,*I*). All examined brainstem areas—but not the cortical regions—expressed VGluT3 mRNA (Extended Data Table to Fig. 5-3 and 5-4), indicating possible clinical relevance of our study.

## Discussion

We examined the role of VGluT3+ neurons in social behavior, and—as possible confounding factors—in mobility, anxiety-like behavior and short-term memory in mice. For the short summary of the main findings, see Table 1.

**Table 1.** Summary of the main findings

Experiment		Knock-out	MRR excitation	MRR inhibition
Mobility	OF	Ø	Ø	↓
	EPM	Ø	Ø	Ø
	Y-maze	Ø	Ø	Ø
Anxiety	OF	↑	Ø	Ø
	EPM	↑	↓	Ø
Social behavior	Social interest	↑ (sociability, tendency in SDT)	↓ (indirect during sociability)	↑ (in SDT)
	Friendly behavior	Ø	Ø	↓ in aggressive context (RIT)
	Aggressive behavior	↑ in anxiogenic context (SIT)	Ø	Ø
Memory	SDT	↓ 24 h SDT	Ø	Ø
	Y-maze	Ø	Ø	Ø
c-Fos activity		↓ in anterior cingulate, infralimbic cortex and medial septum	↑ in the MRR	-

The results are presented in comparison with their respective control groups: that is, VGluT3 wild-type littermates for knock-out mice and control group (expressing only RFP in the VGluT3+ neurons in the MRR) for the excitatory (Gq DREADD + RFP) and inhibitory (Gi DREADD + RFP) chemogenetically manipulated groups. Ø, no difference compared with respective control group; -, not applicable; DREADD, designer receptors exclusively activated by designer drugs; EPM, elevated plus maze; MRR, median raphe region; OF, open field; RFP, red fluorescent protein; RIT, resident intruder test; SDT, social discrimination test; SIT, social interaction test; VGluT3, vesicular glutamate transporter 3.

During previous studies, the complete lack of VGluT3 resulted in deafness (Seal et al., 2008), highly anxious behavior phenotype (Amilhon et al., 2010; Balazsfi et al., 2016; Sakae et al., 2019), altered fear, and stress reactivity (Horvath et al., 2018; Balazsfi et al., 2018a). KO mice proved to be hyperactive, which is circadian dependent and only appears during their active, dark period (Gras et al., 2008; Divito et al., 2015). However, during short observations in a new, aversive (lighted) environment, they might move even less than the controls (Horvath et al., 2018; Balazsfi et al., 2018a; Fazekas et al., 2019; Sakae et al., 2019; de Almeida et al., 2023). Compared with our previous studies (Balazsfi et al., 2018a; Fazekas et al., 2019), in this current project the experiments were done during active period. This could possibly explain why we found no hypoactivity, and the shortness of observation (5 min compared with, for example, 5 h; Gras et al., 2008) could stand behind the undetectability of hyperactivity. Nevertheless, this experimental set-up further confirms the circadian-dependent role of VGluT3 in locomotion.

It is already well accepted in the literature that the constitutive lack of VGluT3 throughout the lifetime of mice results in a highly anxious behavioral phenotype (Amilhon et al., 2010; Balazsfi et al., 2016; Sakae et al., 2019). Accordingly, we showed on EPM that VGluT3 KO mice also exhibited less risk assessment behavior. Moreover, even though OF was conducted in dark, less anxiogenic context, KO animals still preferred the periphery.

In the sociability test, KO animals proved to be more social, which we failed to observe in our previous study (Fazekas et al., 2019). This could be explained by the previously mentioned circadian difference, as well as by slight difference in the protocol. Interestingly, the KO animals showed aggressive behavior only during SIT, which might be explained by the protocol. Although the habituation to the context of SIT is aimed to reduce aggression, the test was conducted in an anxiogenic (lighted) environment, which could have facilitated aggression in the sensitive KO mice, which is known to exhibit high anxiety-like behavior (Amilhon et al., 2010). In accordance with the literature (Amilhon et al., 2010), in RIT there was no significant change in their behavior, further supporting the anxiety-induced nature of aggression during SIT.

Another group linked decreased VGluT3 protein expression to impaired memory (Cheng et al., 2011). We also found KO mice to be worse in certain aspects of learning, such as working memory and cognitive flexibility (Fazekas et al., 2019). A new study on them confirmed their proper learning and recognition memory ability but impaired pattern separation after aversive stimuli (de Almeida et al., 2023). In the SDT, the VGluT3 KO animals had impaired long-term (24 h) social memory. Similarly, in our previous study VGluT3 KO mice showed inadequate long-term (24 h) social memory in the three-chamber sociability test. However, in the Y-maze, which measures working memory, we failed to observe the previously detected impairment (Fazekas et al., 2019). Since the lack of difference was observed during the active phase without hypolocomotion in the present study, but a previous one showed marked hypolocomotion during their inactive phase (de Almeida et al., 2023), this indicates a major sensitivity of these tests to environmental factors, as well as suggest that the KO-induced changes are subtle and can be easily compensated.

Interestingly, social behavior elicited lower neuronal activation in KO than in WT on relevant brain areas (infralimbic cortex and the medial septum). These are projection target of MRR (Leranth and Vertes, 1999; Vertes et al., 1999; McKenna and Vertes, 2001; Szonyi et al., 2016) indicating that MRR might be a key hub in VGluT3-influenced social behavior. Therefore, we conducted an MRR-focused analysis using VGluT3-Cre mice.

The activity of MRR has been linked to locomotion in numerous occasions. Stimulation of the MRR resulted in a decreased locomotion and a long-term fear memory formation (Bland et al., 2016; Balazsfi et al., 2017). By using different pharmacons (Shim et al., 2014), the effect of MRR on locomotion was partly dopamine receptor (D2) dependent and partly independent. According to our results, the later pathway potentially includes VGluT3+ neuronal (in)activity. However, stimulation was ineffective and the locomotor effect was not detected in all tests. Interestingly, when all VGluT3-positive neurons of the body were excited with chemogenetics, hypolocomotion was detected in the light phase of mice (Kljakic et al., 2022). Based on these and our results, the role of VGluT3-positive neurons in locomotion is complex and MRR is just one brain area which participates in this role.

Anxiety-like behavior is strongly tied to serotonin, which is also present in the MRR. By using mRNA interference specific to the serotonin transporter in the MRR, anxiety-like behavior was increased on EPM (Verheij et al., 2018). In contrast, in our study excitation of VGluT3+ cell of MRR decreased anxiety. This is in line with the increased anxiety of KO mice and can be explained by the vesicle loading role of VGluT3 (Amilhon et al., 2010). Thus, we might assume that excitation of MRR-VGluT3 neurons increased serotonin loading, thereby reducing anxiety. However, this effect is not robust as the phenomenon was not observable in OF and inhibition was ineffective.

As of its role in social behavior, optogenetic stimulation of the whole MRR decreased aggression in the SIT paradigm, which was accompanied by not only increased levels of serotonin but also glutamate in the mPFC (Balazsfi et al., 2018b). In our present experiment, the decreased time spent with the conspecific and the increased time spent with other behavior during the sociability test in the excitatory group indicate a decreased social interest. However, inhibition increased social interest only 1 d after manipulation, during the SDT. Additionally, inhibition affected friendly social behavior in a context-specific manner: in the anxiogenic SIT tended to increase it, while in RIT decreased it. However, despite our expectations we were unable to find any other major role of MRR-VGluT3+ cells in social behavior.

MRR has been implicated in memory numerous times. It has an effect on both theta oscillation (Viana Di Prisco et al., 2002; Bland et al., 2016) and ripple activity of the hippocampus (Wang et al., 2015), known elementary phenomena of memory formation. Moreover, it is known that MRR sends nonserotoninergic projections to the medial septum and hippocampus (Aznar et al., 2004; Fortin-Houde et al., 2023), key brain areas of learning and memory (Tsanov, 2018; Jin et al., 2020; Wu et al., 2021). As previously mentioned, excitation of the MRR elicited a fear memory without actual aversive stimulus, while inhibition of the nucleus prevented the memory formation (Balazsfi et al., 2017). Based on these results, it is expected that MRR-VGluT3+ neurons play a role in some sort of learning and/or memory formation processes. Despite this, in the two memory-related experiments (SDT and Y-maze), we failed to identify any effect. Whether they regulate more complex processes still need to be investigated.

In the MRR, the increase in c-Fos-positive neurons was significant after injection, proving two major points: (1) despite the control animals being injected as well, there is no neuronal activation by CNO and (2) the expressed excitatory DREADD receptors were functional.

Interestingly, the excitation and inhibition of the MRR-VGluT3+ neurons did not yield complementary results. However, we must take into consideration that the used DREADDs activated-inhibited different pathways (*G_q_* vs *G_i_*), which could have caused the different outcomes. Moreover, it has been shown that the MRR-VGluT3+ cells are not homogenous. Unfortunately, with chemogenetics we are unable to differentiate between these cells and it is possible that we have seen the interaction of the activity of these cells.

The expression pattern of VGluT3 in the brain is mostly known from rodent studies. In the brainstem, abundant VGluT3 mRNA and protein levels were found in the dorsal and median raphe nuclei, but only punctate pattern in the pontine nuclei (Schafer et al., 2002; Nakamura et al., 2005; Stornetta et al., 2005). Guo et al. (2005) showed VGluT3-positive cells in the cat parabrachial nucleus. Vigneault et al. (2015) conducted in situ hybridization and immunoautoradiography to detect mRNA and protein levels, respectively, of all three types of VGluTs in the human brain on selected brain regions. However, the only brainstem regions they examined were the collective raphe nuclei, highlighting the dorsal raphe and the pons area. In accordance with the literature, they found abundant VGluT3 mRNA and protein expression in the dorsal raphe; however, in the rest of the pons, it was detected only on protein level. By using RT-qPCR on human brain samples, we confirmed the presence of VGluT3 in human brainstem on the mRNA level. Midbrain raphe nuclei, pontine raphe nuclei, pontine reticular formation, and lateral parabrachial nucleus all showed detectable transcripts, while the control cortical samples did not. This is in accordance with the literature on rodents (Fremeau et al., 2002; Gras et al., 2002; Schafer et al., 2002; Herzog et al., 2004; Guo et al., 2005; Stornetta et al., 2005; Hioki et al., 2010).

In summary, our experiments showed that the total ablation of the VGluT3 gene shifted the behavior to be socially more active. However, the fact that the KO animal showed increased aggression only during the anxiogenic SIT test, but not during the RIT, suggests that the aggression of these animals was induced by anxiety. We further confirmed the highly anxious phenotype and an impairment in long-term social memory in the VGluT3 KO animals. In the case of VGluT3+ MRR neurons, we found that they play a role in locomotion and long-term social interest. Although in our case it was not possible to differentiate between the VGluT3+ only and VGluT3+/5-HT+ neurons of the MRR, it was demonstrated that either one or both subpopulations can contribute to the anxiolytic effect of MRR but has subtle effect on social behavior.

## Data Availability

The datasets generated and/or analyzed during the current study are available from the corresponding author on reasonable request.

## Synthesis

Reviewing Editor: Sam Golden, The University of Washington Decisions are customarily a result of the Reviewing Editor and the peer reviewers coming together and discussing their recommendations until a consensus is reached. When revisions are invited, a fact-based synthesis statement explaining their decision and outlining what is needed to prepare a revision will be listed below. The following reviewer(s) agreed to reveal their identity: Aditya Singh. Note: If this manuscript was transferred from JNeurosci and a decision was made to accept the manuscript without peer review, a brief statement to this effect will instead be what is listed below.

Dear Dr. Zelena,

Your manuscript has been reviewed by two experts in the field. In summary, both reviewers recognize the manuscript's contribution to understanding the role of VGlutT3+ neurons in social behavior but advise revisions for clarity, presentation, and methodological details. The manuscript would benefit from a thorough review to address these concerns, enhancing its overall quality and scientific rigor. Upon receipt of a point-by-point rebuttal and revised manuscript, a final editorial decision will be made. Below you will find a synthesis of the reviewer comments, followed by their specific reviews.

Reviewer 1 acknowledges that the study confirms some previously established roles of these neurons while also presenting new findings, particularly the impact of the dark cycle of the circadian rhythm on behavior. However, the reviewer notes inconsistencies and marginal results, particularly in the context of chemogenetic activation or deactivation. The reviewer suggests that the manuscript advances our understanding of VGlutT3 and median raphe neurons in sociability and other behaviors, but also highlights several areas for improvement. These include the need for corrections for multiple t-tests in the c-fos studies, clearer descriptions of results and outcome variables, and clarification on the exclusion criteria for outliers.

Reviewer 2 commends the manuscript for providing interesting data on the behavior and neural underpinnings in VGluT3 systemic and conditional cre-based KO mice, noting its overall good writing quality. However, this reviewer also suggests numerous specific improvements, mainly concerning clarity, presentation, and grammar. These suggestions include rephrasing various lines for clearer understanding, improving the graphics and color scheme of Figure 1, clarifying experimental details such as animal numbers in certain conditions, and rephrasing sentences to enhance clarity and relevance. There are also recommendations to correct spelling errors and to provide more specific and detailed explanations in certain sections of the manuscript.

Reviewer 1:

This manuscript investigates the role of VGlutT3+ neurons in social and other related forms of behavior. Prior work has established a range of behaviors in KO animals and this manuscript confirms these roles to some extend while also showing some differences due to being run during the dark cycle of the circadian rhythm. It also utilizes viral methods to target these cells specifically in the median raphe. Several differences were observed in the case of chemogenetic activation or deactivation in relation to KO animals although the results often were inconsistent or marginal. Overall however, it does advance knowledge of the role of both the VGlutT3 and median raphe neurons in sociability and other behaviors.

A few concerns are noted below in order to improve the overall manuscript.

No corrections were done for multiple t tests in the case of c-fos studies where multiple comparisons were made. Due to inclusion of marginal significance a priori, adjustment of significance when looking at multiple regions may alter some interpretation of results and should be included.

The methods section mentions exclusion criteria for outliers in the data but it is not mentioned when this occurred in any of the results section.

At times descriptions of results are somewhat vague as to outcome variables or are confusing. For example on line 376 it says "During object habituation there was a marginal effect of choice (F(1,32)=3.990,p=0.055), without significant post-hoc effects." Or on line 384 etc when describing "other" behavior. Please be more descriptive of the outcome variables being measured and how they relate to manipulations throughout the results sections. I found that I had to refer back to the methods several times to make sense of descriptions in the results.

Reviewer 2:

The manuscript provides interesting data to compare the behavior and its neural underpinnings in VGluT3 systemic and conditional cre-based KO mice. Overall, well written, some improvement required in framing results, presentation, and grammar for some of the sentences.

I have following comments:

- Presentation in Figure 1 should be improved. Use a mild color scheme, flow chart can be designed with improved graphics, and Panels for Figure 1B can be reduced in size.

-Line 33 - Rephrase the first sentence as "...its dysfunctions impact several pathological conditions".

-Line 34 - replace the word "unquestionable" with "undeniable" or "understood".

-Line 42-43 - remove the words "locomotor" and "anxiety" and "memory". (OF is also a test for anxiety.)

Rephrase line 46-47 as, "For KO animals, social interaction induced lower cell activation in the..."

-Line 52 - MMR? Should it be MRR?

-Line 54 - Rephrase as, "The presence of VGlut3 mRNA on corresponding human brain areas suggests clinical relevance".

-Line 56 - A word is missing after subcortical (areas/regions?)

-Line 70 - Replace "has already begun" with " is ongoing".

-Line 78-79 - Remove word "circa" and rephrase as, "However, only 5.742% of the MRR neurons have been shown to express...."

-Line 80 - Replace "expresses" as "express".

-Line 100 - Rephrase the last part of the sentence, "...lifelong and only negative influence". Not clear what the author is trying to convey.

-Line 101-103 - Correct spelling - Neurones to Neurons. Rephrase the sentences "...neurones might..." with "...neurons would shed new light on the role of this specific subpopulation, with the advantage of short-term influence on both stimulation and inhibition. The comparison of the two models would provide deeper insight on the VGlut3 function."

-Line 113 and 143- Replace "half an hour" with "30 min".

-Line 124 - Were only N=3 animals manipulated for excitatory condition? This doesn't match the figures which show a higher number of animals in excitatory condition (Green bar plot). Clarify.

-Line 187 and 189 - Replace "Aquarium" with "chamber"

-Line 206 - Are any visual cues used for the y-maze?

-Line 234 - Correct spelling "Secunder" - "Secondary"

-Line 285 - Clarify the sentence - what do authors refer to by "both series". Also, what was ANOVA not used for comparing cFos positive cell numbers?

-Line 303 - replace "compartments" with "zones".

-Line 304 - remove the word "either" at the end of the sentence.

-Line 328 - clarify sentence "...percentage of time spent with" whom? Conspecific or familiar or novel mouse?

-Line 345 - rephrase "but the KO group did" as " unlike the KO group".

-Line 350 - mention y-maze in the sentence.

-Line 361 - Replace "effect" with "affect"

-Line 379 - Replace "meaning that" with "indicating"

-Line 387 - What is adequate? How is adequate defined for each group?

-Line 401 - Remove words "of this"

-Line 405 - add "manipulation in both frequencies of interaction"...

-Line 442 - Replace "that" with "than"

-Line 470 - Add the word "show" after KO mice.

-Line 473-473 - Rephrase the sentence remove repetition of authors name in citation and in the text.

-Line 498 - Replace "be" with "by"

-Line 499 - Correct spelling "neurones"

-Line 550 - clarify and rephrase - what do authors mean by "..result in an inadequate appearance of aggression in anxiogenic contexts"? Is aggression expected in anxiogenic contexts and how is adequacy of aggression in anxiogenic contexts estimated?

## Author Response

Reviewer 1.

Thank you for your work and suggestions. According to your review, we corrected our manuscript.

1. No corrections were done for multiple t tests in the case of c-fos studies where multiple comparisons were made. Due to inclusion of marginal significance a priori, adjustment of significance when looking at multiple regions may alter some interpretation of results and should be included.

We are sorry for the confusion regarding the c-Fos result analysis. Indeed, there were no correction for them, as the immunohistological staining against c-Fos was done separately for each region due to the high number of brain slices. As per recommendation, we utilised Benjamini-Hochberg (FDR, false discovery rate) post-hoc correction, and thus the following modifications were made in the manuscript:

Materials and methods, Statistical analysis: "The c-Fos positive cell counting was done for every brain region separately, as staining was conducted also separately. Therefore, the groups were also compared with t-test (WT-KO: effect of genotype; VGluT3-Cre control-excitatory: effect of manipulation). However, post-hoc correction for multiple comparisons was also done by Benjamini-Hochberg correction (FDR, false discovery rate)." Results, VGluT3 KO studies, neuronal activity: "There was a significant decrease in the number of c-Fos positive cells in the medial septum (t(11)= 4.199; p=0.001) and infralimbic cortex (t(11)=3.402; p=0.006) of VGluT3 KO mice compared to WT. Other brain areas did not show significant difference after FDR correction (Fig.5A-D; Extended Data to Figure 5-1.)." Discussion: "Interestingly, social behaviour elicited lower neuronal activation in KO than in WT on relevant brain areas (infralimbic cortex and the medial septum). These are projection target of MRR (Leranth and Vertes, 1999; Vertes et al., 1999; McKenna and Vertes, 2001; Szonyi et al., 2016) indicating that MRR might be a key hub in VGluT3-influenced social behaviour." Extended data legends, Extended Data to Figure 5-1.: "Benjamini-Hochberg (FDR, false discovery rate) post-hoc correction was used, thus a p-value was considered significant from 0.016." Extended data legends, Extended Data to Figure 5-2.: "Benjamini-Hochberg (FDR, false discovery rate) post-hoc correction was used, thus a p-value was considered significant from 0.008." Extended Data, Extended Data to Figure 5-1.: results were updated according to corrections.

2. The methods section mentions exclusion criteria for outliers in the data but it is not mentioned when this occurred in any of the results section.

As per request, the following section to the Statistical analysis was added for clarification: "This outlier analysis revealed uncharacteristic behaviour resulted in the exclusion of the following number of animals: 1 WT mouse in the OF test; 3 WT mice in the Y-maze test; 1 WT and 1 KO mice in sociability and social discrimination tests. Similarly, in the case of VGluT3-Cre mice the following number of animals were excluded: 1 mouse in the inhibitory group in the Y-maze test; 2 mice in the excitatory group during sociability test - object habituation phase, out of which 1 had to be also excluded from the rest of the experiment (sociability phase, social discrimination test) as well. In the SIT and the EPM test, 1-1 mouse from the excitatory group was excluded, respectively. Finally, 2 mice (1 control and 1 inhibitory) had to be removed from the RIT analysis." 3. At times descriptions of results are somewhat vague as to outcome variables or are confusing. For example on line 376 it says "During object habituation there was a marginal effect of choice (F(1,32)=3.990,p=0.055), without significant post-hoc effects." Or on line 384 etc when describing "other" behavior. Please be more descriptive of the outcome variables being measured and how they relate to manipulations throughout the results sections. I found that I had to refer back to the methods several times to make sense of descriptions in the results.

The results part were corrected accordingly to the comment, and clarifications were added, such as " 'other', non-social behaviour" (line 411), or better explaining statistical results, like "During object habituation there was a marginal effect of choice (F(1,32)=3.990,p=0.055), without significant post-hoc effects between the two empty cages (Extended Data to Figure 3-6.), and thus we did not apply correction for side preference during subsequent analysis" (line 394-396).

- Reviewer 2.

Thank you for your thorough work and suggestions. We rewrote our review according to your review. Your comments regarding grammar and phrasing (Line 33, 34, 42-43, 46-47, 52, 54, 56, 70, 78-79, 80,101-103, 113,143, 187, 189, 234, 303, 304, 345, 350, 361, 379, 401, 405, 442, 470, 473, 498 and 499) were all corrected according to suggestions. Figure 1 was revised to show less vivid colours and was re-sized.

Regarding more detailed answers:

1. Line 100 - Rephrase the last part of the sentence, "...lifelong and only negative influence". Not clear what the author is trying to convey.

Sentence was specified and rephrased to reflect the original intention: "With the usage of VGluT3 knockout (KO) mice the global role of this transporter could be revealed, however, life-long, constitutive lack of a gene product could result in compensatory mechanisms, ultimately masking behavioural effects." 2. Line 124 - Were only N=3 animals manipulated for excitatory condition? This doesn't match the figures which show a higher number of animals in excitatory condition (Green bar plot). Clarify.

We are sorry for the confounding statements. For the behaviour, N=13 was the number of animals in the excitatory group, and ultimately depicted on the figures (green bar plots). In the experiments regarding c-Fos expression, the number of animals in the excitatory group was N=8, and the results are shown in Extended Data Figure 5-1.

Thus, the manuscript was corrected accordingly: "C-Fos activation was studied in a different batch of animals from those used in the behavioural studies. Wild-type (N=5) and VGluT3 KO (N=8) mice were perfused 90-min after a social interaction test." 3. Line 206 - Are any visual cues used for the y-maze? No, there were no visual cues in the Y-maze. The following sentence was added for clarification: "No extra external spatial cues were used during the experiment." 4. Line 285 - Clarify the sentence - what do authors refer to by "both series". Also, what was ANOVA not used for comparing cFos positive cell numbers? We are sorry for the confusing phrasing. As the c-Fos staining were done separately between the different brain regions, resulting in different staining intensity, and only two groups were investigated (WT-KO and VGluT3-Cre control-excitatory), thus, individual t-tests were done for each area. As this was a concern of the other reviewer, we revisited our data and applied Benjamini-Hochberg (FDR, false discovery rate) post-hoc correction, and the detailed changes made in the manuscript are highlighted with yellow and can be found in the answer to Reviewer 1.

5. Line 328 - clarify sentence "...percentage of time spent with" whom? Conspecific or familiar or novel mouse? That phrase was referring to the percentage of time spent with aggressive behaviour. Manuscript was corrected accordingly: "However, there was an increase in the percentage of time (t(19)=-2.182, p=0.042) and frequency of aggressive behaviour (t(19)=-2.762, p=0.012) in KO compared to WT." 6. Line 387 - What is adequate? How is adequate defined for each group? The sentence was corrected to contain more explanation: "All three groups had adequate, higher than 50% SI (control: t(7)=13.670,p=0.001; excitatory: t(11)=7.324,p<0.001; inhibitory: t(14)=6.658,p<0.001), indicating preference for social stimulus, without differences between the groups (Extended Data Figure 3-7.)." 7. Line 550 - clarify and rephrase - what do authors mean by "..result in an inadequate appearance of aggression in anxiogenic contexts"? Is aggression expected in anxiogenic contexts and how is adequacy of aggression in anxiogenic contexts estimated? The section was clarified to reflect the original meaning better: "Although the fact that the KO animal showed increased aggression only during the anxiogenic SIT test, but not during the RIT, suggests that the aggression of these animals was induced by anxiety."
