# Supplementary material for: The Role of Vesicular Glutamate Transporter Type 3 in Social Behavior, with a Focus on the Median Raphe Region
Source: eNeuro. 2024 Jun 3;11(6):ENEURO.0332-23.2024. doi: 10.1523/ENEURO.0332-23.2024 (PMC11154661; doi:10.1523/ENEURO.0332-23.2024)
Supplement: Figure 2-1 — Results of open field test - VGluT3 WT-KO animals. Degree of freedom (df) for the two-sample t-tests was 18. Data are expressed as mean ± SEM. WT: wild-type; KO: knock-out. ** p < 0.01 vs WT. Download Figure 2-1, DOCX file. [file eneuro-11-ENEURO.0332-23.2024-s001.docx]

**Extended Data Table to Figure 2-1.** **Results of open field test - VGluT3 WT-KO animals.**

| **Genotype** | | **WT  (N=9)** | **KO  (N=11)** | **t-value** | **p-value** |
| --- | --- | --- | --- | --- | --- |
| **Distance moved (cm)** | | 2244.798$\pm$100.006 | 2251.459$\pm$225.590 | -0.025 | 0.980 |
| **Frequency** | **Centrum** | 49.000$\pm$3.768 | 46.273$\pm$5.128 | 0.411 | 0.685 |
| **Time %)** | **Centrum** | 37.858$\pm$2.821 | 28.014$\pm$1.932  ****** | 2.963 | 0.008 |
