# Supplementary material for: The Role of Vesicular Glutamate Transporter Type 3 in Social Behavior, with a Focus on the Median Raphe Region
Source: eNeuro. 2024 Jun 3;11(6):ENEURO.0332-23.2024. doi: 10.1523/ENEURO.0332-23.2024 (PMC11154661; doi:10.1523/ENEURO.0332-23.2024)
Supplement: Figure 2-2 — Results of elevated plus-maze test - VGluT3 WT-KO animals. WT-KO: Degree of freedom (df) for the two-sample t-test (all parameters) is 19. Marginal effects are in brackets (). Data are expressed in mean ± SEM. WT: wild-type; KO: knock-out; RA: risk assessment; SAP: stretched attend posture. * p < 0.05 vs WT. Download Figure 2-2, DOCX file. [file eneuro-11-ENEURO.0332-23.2024-s002.docx]

**Extended Data Table to Figure 2-2. Results of elevated plus-maze test - VGluT3 WT-KO animals.**

| **Genotype** | | **WT (N=10)** | **KO (N=11)** | **t-value** | **p-value** |
| --- | --- | --- | --- | --- | --- |
| **Frequency** | **Closed arm** | 13.500$\pm$1.827 | 13.818$\pm$1.872 | -0.121 | 0.905 |
|  | **Centrum** | 21.500$\pm$3.208 | 18.636$\pm$2.813 | 0.674 | 0.508 |
|  | **Open arm** | 8.000$\pm$1.640 | 4.818$\pm$1.143 | 1.616 | 0.122 |
| **Time (%)** | **Closed arm** | 67.930$\pm$5.198 | 77.027$\pm$4.802 | -1.288 | 0.213 |
|  | **Centrum** | 14.270$\pm$1.867 | 13.491$\pm$2.023 | 0.281 | 0.782 |
|  | **Open arm** | 17.040$\pm$3.958 | 8.791$\pm$2.952 | 1.691 | 0.107 |
| **Open/total (%)** | | 34.746$\pm$4.308 | 23.520$\pm$3.576***** | 2.019 | (0.058) |
| **RA**  **frequency** | **Head dipping** | 13.300$\pm$1.856 | 10.091$\pm$2.274 | 1.080 | 0.294 |
|  | **SAP** | 34.300$\pm$2.486 | 27.909$\pm$3.767 | 1.386 | 0.182 |
|  | **Rearing** | 19.900$\pm$2.923 | 11.091$\pm$1.745* | 2.645 | 0.016 |
| **Grooming** | | 1.800$\pm$0.249 | 2.273$\pm$0.488 | -0.836 | 0.413 |
