# Supplementary material for: The Role of Vesicular Glutamate Transporter Type 3 in Social Behavior, with a Focus on the Median Raphe Region
Source: eNeuro. 2024 Jun 3;11(6):ENEURO.0332-23.2024. doi: 10.1523/ENEURO.0332-23.2024 (PMC11154661; doi:10.1523/ENEURO.0332-23.2024)
Supplement: Figure 2-3 — Results of open field test - VGluT3-Cre animals. Degree of freedom (df) for the one-way ANOVA was (2,32). Marginal effects are in brackets (). Data are expressed as mean ± SEM. Download Figure 2-3, DOCX file. [file eneuro-11-ENEURO.0332-23.2024-s003.docx]

**Extended Data Table to Figure 2-3.** **Results of open field test - VGluT3-Cre animals.**

| **DREADD type** | | **Control (N=8)** | **Excitatory (N=13)** | **Inhibitory (N=14)** | **F-value** | **p-value** |
| --- | --- | --- | --- | --- | --- | --- |
| **Distance moved (cm)** | | 2847.871$\pm$219.809 | 2892.461$\pm$ 160.103 | 2422.187$\pm$ 115.725 | 3.038 | (0.062) |
| **Frequency** | **Centrum** | 59.125$\pm$ 6.278 | 59.308$\pm$ 3.249 | 56.933$\pm$ 3.521 | 0.119 | 0.888 |
| **Time (%)** | **Centrum** | 39.201$\pm$ 1.964 | 38.366$\pm$ 3.762 | 40.142$\pm$ 2.742 | 0.091 | 0.913 |
