# Supplementary material for: The Role of Vesicular Glutamate Transporter Type 3 in Social Behavior, with a Focus on the Median Raphe Region
Source: eNeuro. 2024 Jun 3;11(6):ENEURO.0332-23.2024. doi: 10.1523/ENEURO.0332-23.2024 (PMC11154661; doi:10.1523/ENEURO.0332-23.2024)
Supplement: Figure 2-4 — Results of elevated plus-maze test – VGluT3-Cre animals. Degree of freedom (df) for the one-way ANOVA for the frequency and time (%) spent in different zones is (2,32), while for the risk assessment behaviour is (2,31). Marginal effects are in brackets (). Data are expressed in mean ± SEM. RA: risk assessment; SAP: stretched attend posture. == p < 0.01 vs control; & p < 0.05 vs inhibitory Download Figure 2-4, DOCX file. [file eneuro-11-ENEURO.0332-23.2024-s004.docx]

**Extended Data Table to Figure 2-4. Results of elevated plus-maze test – VGluT3-Cre animals.**

| **DREADD type** | | **Control (N=8)** | **Excitatory (N=12)** | **Inhibitory (N=15)** | **F-value** | **p-value** |
| --- | --- | --- | --- | --- | --- | --- |
| **Frequency** | **Closed arm** | 20.375±1.546 | 16.750±1.349 | 17.133±1.338 | 1.554 | 0.227 |
|  | **Centrum** | 27.250±2.202 | 26.250±1.737 | 24.800±1.818 | 0.400 | 0.673 |
|  | **Open arm** | 6.875±1.187 | 9.333±1.061 | 7.533±0.689 | 1.698 | 0.199 |
| **Time (%)** | **Closed arm** | 62.888±4.926 | 51.158±4.903 | 58.493±3.327 | 1.680 | 0.202 |
|  | **Centrum** | 18.350±3.015 | 17.900±1.880 | 16.793±1.569 | 0.162 | 0.851 |
|  | **Open arm** | 17.800±5.088 | 30.033±5.567 | 23.847±3.707 | 1.391 | 0.263 |
| **Open/total (%)** | | 24.521±2.861 | 35.965±3.440 **==** | 30.638±1.727 | 3.828 | 0.032 |
| **RA**  **frequency** | **Head dipping** | 10.125±2.531 | 18.000±2.841 **=&** | 10.867±1.588 | 3.572 | 0.040 |
|  | **SAP** | 24.875±4.340 | 35.545±4.509 | 38.600±6.375 | 1.317 | 0.282 |
|  | **Rearing** | 8.375±2.104 | 12.636±1.521 | 14.533±1.701 | 2.769 | (0.078) |
| **Grooming** | | 2.000±0.267 | 1.273±0.333 | 1.267±0.228 | 1.868 | 0.171 |
