# Supplementary material for: The Role of Vesicular Glutamate Transporter Type 3 in Social Behavior, with a Focus on the Median Raphe Region
Source: eNeuro. 2024 Jun 3;11(6):ENEURO.0332-23.2024. doi: 10.1523/ENEURO.0332-23.2024 (PMC11154661; doi:10.1523/ENEURO.0332-23.2024)
Supplement: Figure 3-1 — Results of sociability test – object habituation phase – VGluT3 WT-KO animals. Degree of freedom (df) for the two-sample t-test (frequency and time [%] of ‘other’ behaviour) was 17. Degree of freedom in the repeated-measures ANOVA (frequency and time [%] of left vs right cage) was (1,17) for all effects. Data are expressed in mean ± SEM. WT: wild-type; KO: knock-out. * p < 0.05 vs WT. Download Figure 3-1, DOCX file. [file eneuro-11-ENEURO.0332-23.2024-s005.docx]

**Extended Data Table to Figure 3-1. Results of sociability test – object habituation phase – VGluT3 WT-KO animals.**

| **Genotype** | | **WT (N=9)** | **KO  (N=10)** | **F- or t-value** | **p-value** |
| --- | --- | --- | --- | --- | --- |
| **Frequency** | **Left cage** | 18.889$\pm$1.767 | 18.400$\pm$2.023 | Genotype:  0.000  Choice:  0.553  Genotype$\times$Choice:  0.109 | 0.996  0.467  0.746 |
|  | **Right cage** | 17.333$\pm$2.211 | 17.800$\pm$1.769 |  |  |
|  | **‘Other’ behaviour** | 37.111$\pm$3.276 | 37.100$\pm$3.291 | 0.002 | 0.998 |
| **Time (%)** | **Left cage** | 7.652$\pm$1.036 | 9.685$\pm$0.761 | Genotype:  4.378  Choice:  0.098  Genotype$\times$Choice:  0.469 | 0.052  0.758  0.502 |
|  | **Right cage** | 6.954$\pm$1.184 | 9.945$\pm$0.945 |  |  |
|  | **‘Other’ behaviour** | 85.394$\pm$2.026 | 80.370$\pm$1.370***** | 2.092 | 0.052 |
