# Supplementary material for: The Role of Vesicular Glutamate Transporter Type 3 in Social Behavior, with a Focus on the Median Raphe Region
Source: eNeuro. 2024 Jun 3;11(6):ENEURO.0332-23.2024. doi: 10.1523/ENEURO.0332-23.2024 (PMC11154661; doi:10.1523/ENEURO.0332-23.2024)
Supplement: Figure 3-2 — Results of sociability test -sociability phase – VGluT3 WT-KO animals. Degree of freedom (df) for the two-sample t-test (frequency and time [%] of ‘other’ behaviour; SI) is 18. Degree of freedom in the repeated-measures ANOVA (frequency and time [%] of left vs right cage) is (1,18) for all effects. Marginal effects are in brackets (). Data are expressed in mean ± SEM. WT: wild-type; KO: knock-out; SI: sociability index. ## p < 0.01 vs cage; ** p < 0.01, * p < 0.05 vs WT; $$ p < 0.01 vs random 50. Download Figure 3-2, DOCX file. [file eneuro-11-ENEURO.0332-23.2024-s006.docx]

**Extended Data Table to Figure 3-2. Results of sociability test -sociability phase – VGluT3 WT-KO animals.**

| **Genotype** | | **WT (N=10)** | **KO (N=11)** | **F- or t-value** | **p-value** |
| --- | --- | --- | --- | --- | --- |
| **Frequency** | **Mouse** | 18.111$\pm$1.859 | 22.818$\pm$1.853 | Genotype:  0.871  Choice:  16.503  Genotype$\times$Choice:  3.327 | 0.363  0.000  (0.085) |
|  | **Cage** | 15.000$\pm$2.034 | 14.636$\pm$1.870 |  |  |
|  | **‘Other’ behaviour** | 32.556$\pm$3.105 | 36.091$\pm$2.859 | -0.836 | 0.414 |
| **Time (%)** | **Mouse** | 24.341$\pm$5.785  **##** | 40.336$\pm$3.514  **## **** | Genotype:  6.066  Choice:  47.541  Genotype$\times$Choice:  5.246 | 0.024  0.000  0.034 |
|  | **Cage** | 8.463$\pm$1.311 | 8.661$\pm$1.180 |  |  |
|  | **‘Other’ behaviour** | 67.196$\pm$6.043 | 51.003$\pm$3.333 | 2.463 | 0.024 |
| **SI** | | 69.263$\pm$5.246**$$** | 81.236$\pm$2.938**$$ *** | -2.087 | 0.051 |
