# Supplementary material for: The Role of Vesicular Glutamate Transporter Type 3 in Social Behavior, with a Focus on the Median Raphe Region
Source: eNeuro. 2024 Jun 3;11(6):ENEURO.0332-23.2024. doi: 10.1523/ENEURO.0332-23.2024 (PMC11154661; doi:10.1523/ENEURO.0332-23.2024)
Supplement: Figure 3-3 — Results of social interaction test – VGluT3 WT-KO animals. Degree of freedom (df) for the two-sample t-test (all parameters) is 19. Marginal effects are in brackets (). Data are expressed in mean ± SEM. WT: wild-type; KO: knock-out. * p < 0.05 vs WT. Download Figure 3-3, DOCX file. [file eneuro-11-ENEURO.0332-23.2024-s007.docx]

**Extended Data Table to Figure 3-3. Results of social interaction test – VGluT3 WT-KO**

**animals.**

| **Genotype** | | **WT (N=10)** | **KO (N=11)** | **t-value** | **p-value** |
| --- | --- | --- | --- | --- | --- |
| **Frequency** | **Social behaviour** | 42.500$\pm$2.062 | 50.818$\pm$4.274 | -1.697 | 0.106 |
|  | **Aggressive behaviour** | 0.000$\pm$0.000 | 2.818$\pm$0.971***** | -2.762 | 0.012 |
|  | **Defensive behaviour** | 0.000$\pm$0.000 | 1.545$\pm$0.813 | -1.808 | (0.086) |
|  | **‘Other’ behaviour** | 43.400$\pm$2.077 | 52.545$\pm$4.082 | -1.936 | (0.068) |
| **Time (%)** | **Social behaviour** | 28.994$\pm$2.971 | 34.364$\pm$2.923 | -1.286 | 0.214 |
|  | **Aggressive behaviour** | 0.000$\pm$0.000 | 2.673$\pm$1.181***** | -2.182 | 0.042 |
|  | **Defensive behaviour** | 0.000$\pm$0.000 | 0.880$\pm$0.682 | -1.228 | 0.234 |
|  | **‘Other’ behaviour** | 67.706$\pm$4.760 | 62.082$\pm$3.039 | 1.015 | 0.323 |
