# Supplementary material for: The Role of Vesicular Glutamate Transporter Type 3 in Social Behavior, with a Focus on the Median Raphe Region
Source: eNeuro. 2024 Jun 3;11(6):ENEURO.0332-23.2024. doi: 10.1523/ENEURO.0332-23.2024 (PMC11154661; doi:10.1523/ENEURO.0332-23.2024)
Supplement: Figure 3-4 — VGluT3 WT-KO contingency table. The table shows the numbers of animals who exhibited aggressive behaviour in social interaction test, followed by a Pearson Chi-square test. WT: wild-type; KO: knock-out. Download Figure 3-4, DOCX file. [file eneuro-11-ENEURO.0332-23.2024-s008.docx]

**Extended Data Table to Figure 3-4. VGluT3 WT-KO contingency table**.

| **Aggressive behaviour**  **Genotype** | **Yes** | **No** | **Total** |
| --- | --- | --- | --- |
| **WT** | 0 | 10 | 10 |
| **KO** | 6 | 5 | 11 |
| **Total** | 6 | 15 | 21 |
| Pearson Chi-square: 7.636; df=1; p=0.006 | | | |
