# Supplementary material for: The Role of Vesicular Glutamate Transporter Type 3 in Social Behavior, with a Focus on the Median Raphe Region
Source: eNeuro. 2024 Jun 3;11(6):ENEURO.0332-23.2024. doi: 10.1523/ENEURO.0332-23.2024 (PMC11154661; doi:10.1523/ENEURO.0332-23.2024)
Supplement: Figure 3-5 — Results of resident intruder test – VGluT3 WT-KO animals. Degree of freedom (df) for the two-sample t-test (all parameters) is 19. Marginal effects are in brackets (). Data are expressed in mean ± SEM. WT: wild-type; KO: knock-out. == p < 0.01 vs control. Download Figure 3-5, DOCX file. [file eneuro-11-ENEURO.0332-23.2024-s009.docx]

**Extended Data Table to Figure 3-5. Results of resident intruder test – VGluT3 WT-KO animals.**

| **Genotype** | | **WT (N=10)** | **KO (N=11)** | **t-value** | **p-value** |
| --- | --- | --- | --- | --- | --- |
| **Frequency** | **Social behaviour** | 55.700$\pm$3.839 | 56.273$\pm$3.300 | -0.114 | 0.911 |
|  | **Aggressive behaviour** | 6.100$\pm$1.215 | 7.636$\pm$3.566 | -0.392 | 0.700 |
|  | **Defensive behaviour** | 2.900$\pm$1.394 | 0.364$\pm$0.244 | 1.878 | (0.076) |
|  | **‘Other’ behaviour** | 55.000$\pm$3.095 | 57.636$\pm$2.688 | -0.646 | 0.526 |
| **Time (%)** | **Social behaviour** | 35.226$\pm$4.156 | 35.925$\pm$4.000 | -0.121 | 0.905 |
|  | **Aggressive behaviour** | 4.384$\pm$1.577 | 6.566$\pm$3.354 | -0.569 | 0.576 |
|  | **Defensive behaviour** | 3.084$\pm$2.007 | 0.261$\pm$0.208 | 1.470 | 0.158 |
|  | **‘Other’ behaviour** | 57.308$\pm$3.980 | 57.248$\pm$3.829 | -0.646 | 0.526 |
