# Supplementary material for: The Role of Vesicular Glutamate Transporter Type 3 in Social Behavior, with a Focus on the Median Raphe Region
Source: eNeuro. 2024 Jun 3;11(6):ENEURO.0332-23.2024. doi: 10.1523/ENEURO.0332-23.2024 (PMC11154661; doi:10.1523/ENEURO.0332-23.2024)
Supplement: Figure 3-6 — Results of sociability test – object habituation phase – VGluT3-Cre animals. Degree of freedom (df) for the one-way ANOVA (frequency and time [%] of ‘other’ behaviour) was (2,31). Degree of freedom in the repeated-measures ANOVA (frequency and time [%] of left vs right) is (2,31) for the effect of manipulation and manipulation × choice interaction, while (1,32) for the effect of choice. Marginal effects are in brackets (). Data are expressed in mean ± SEM. Download Figure 3-6, DOCX file. [file eneuro-11-ENEURO.0332-23.2024-s010.docx]

**Extended Data Table to Figure 3-6. Results of sociability test – object habituation phase – VGluT3-Cre animals.**

| **DREADD type** | | **Control  (N=8)** | **Excitatory (N=13)** | **Inhibitory  (N=14)** | **F-value** | **p-value** |
| --- | --- | --- | --- | --- | --- | --- |
| **Frequency** | **Left cage** | 27.625$\pm$ 1.580 | 25.364$\pm$ 1.114 | 25.733$\pm$ 1.318 | Manipulation:  0.552  Choice:  3.990  Manipulation $\times$Choice:  0.557 | 0.581  (0.055)  0.579 |
|  | **Right cage** | 28.625$\pm$ 1.679 | 26.636$\pm$ 0.856 | 28.667$\pm$ 1.840 |  |  |
|  | **‘Other’ behaviour** | 57.250$\pm$ 2.889 | 52.818$\pm$ 1.500 | 55.267$\pm$ 2.907 | 0.494 | 0.615 |
| **Time (%)** | **Left cage** | 11.750$\pm$ 1.128 | 9.991$\pm$ 0.754 | 12.307$\pm$ 0.911 | Manipulation:  2.290  Choice:  0.819  Manipulation $\times$Choice:  0.7065 | 0.118  0.372  0.501 |
|  | **Right cage** | 11.600$\pm$ 1.262 | 10.318$\pm$ 0.618 | 13.913$\pm$ 1.584 |  |  |
|  | **‘Other’ behaviour** | 74.963$\pm$ 2.293 | 78.036$\pm$ 1.030 | 72.047$\pm$ 2.311 | 2.312 | 0.116 |
