# Supplementary material for: The Role of Vesicular Glutamate Transporter Type 3 in Social Behavior, with a Focus on the Median Raphe Region
Source: eNeuro. 2024 Jun 3;11(6):ENEURO.0332-23.2024. doi: 10.1523/ENEURO.0332-23.2024 (PMC11154661; doi:10.1523/ENEURO.0332-23.2024)
Supplement: Figure 3-7 — Results of sociability test – sociability phase – VGluT3-Cre animals. Degree of freedom (df) for the one-way ANOVA (frequency and time [%] of ‘other’ behaviour) is (2,32). Degree of freedom in the repeated-measures ANOVA (frequency and time [%] of mouse vs cage) is (2,32) for the effect of manipulation and manipulation × choice interaction, while (1,32) for the effect of choice. Data are expressed in mean ± SEM. SI: sociability index. ## p < 0.01 vs cage; - p < 0.05 vs inhibitory; $ p < 0.05 vs random 50. Download Figure 3-7, DOCX file. [file eneuro-11-ENEURO.0332-23.2024-s011.docx]

**Extended Data Table to Figure 3-7. Results of sociability test – sociability phase – VGluT3-Cre animals.**

| **DREADD type** | | **Control (N=8)** | **Excitatory (N=11)** | **Inhibitory (N=15)** | **F-value** | **p-value** |
| --- | --- | --- | --- | --- | --- | --- |
| **Frequency** | **Mouse** | 27.750± 1.346 | 23.833± 1.114 | 25.400± 2.522 | Manipulation:  1.388  Choice:  33.030  Manipulation$\times$Choice:  0.667 | 0.264  0.000  0.520 |
|  | **Cage** | 19.625± 1.164 | 17.667± 1.233 | 15.667± 1.508 |  |  |
|  | **‘Other’ behaviour** | 47.875± 1.315 | 42.167± 1.957 | 40.400± 3.048 | 1.860 | 0.172 |
| **Time (%)** | **Mouse** | 32.025± 2.030 | 24.467± 2.321 | 35.348± 3.283 | Manipulation:  6.002  Choice:  88.767  Manipulation$\times$Choice:  1.317 | 0.006  0.000  0.282 |
|  | **Cage** | 8.525± 1.078 | 8.067± 0.985 | 11.282± 2.235 |  |  |
|  | **‘Other’ behaviour** | 58.000± 2.448 | 66.083± 2.309**-** | 52.497± 3.447 | 5.580 | 0.008 |
| **SI** | | 78,991± 2,121**$** | 74.367± 3.327**$** | 76.518± 3.983**$** | 0.328 | 0.723 |
