# Supplementary material for: The Role of Vesicular Glutamate Transporter Type 3 in Social Behavior, with a Focus on the Median Raphe Region
Source: eNeuro. 2024 Jun 3;11(6):ENEURO.0332-23.2024. doi: 10.1523/ENEURO.0332-23.2024 (PMC11154661; doi:10.1523/ENEURO.0332-23.2024)
Supplement: Figure 3-8 — Results of social interaction test – VGlut3-Cre animals. Degree of freedom (df) for the one-way ANOVA (all parameters) is (2,32). Marginal effects are in brackets (). Data are expressed in mean ± SEM. = p < 0.05 vs control; @ p < 0.05 vs excitatory Download Figure 3-8, DOCX file. [file eneuro-11-ENEURO.0332-23.2024-s012.docx]

**Extended Data Table to Figure 3-8. Results of social interaction test – VGlut3-Cre animals.**

| **DREADD type** | | **Control (N=8)** | **Excitatory (N=13)** | **Inhibitory (N=15)** | **F-value** | **p-value** |
| --- | --- | --- | --- | --- | --- | --- |
| **Frequency** | **Social behaviour** | 29.000±3.505 | 28.833±2.246 | 35.600±2.099 | 2.702 | (0.082) |
|  | **Aggressive behaviour** | 0.000±0.000 | 0.250±0.250 | 0.000±0.000 | 0.956 | 0.395 |
|  | **Defensive behaviour** | 0.000±0.000 | 0.667±0.667 | 1.467±1.268 | 0.480 | 0.623 |
|  | **‘Other’ behaviour** | 29.750±3.458 | 30.000±2.256 | 37.467±1.662  **= @** | 4.089 | 0.026 |
| **Time (%)** | **Social behaviour** | 9.364±2.704 | 11.758±1.897 | 17.459±3.170 | 2.178 | 0.130 |
|  | **Aggressive behaviour** | 0.000±0.000 | 0.283±0.283 | 0.000±0.000 | 0.956 | 0.395 |
|  | **Defensive behaviour** | 0.000±0.000 | 0.803±0.803 | 1.166±1.110 | 0.331 | 0.721 |
|  | **‘Other’ behaviour** | 90.625±2.701 | 87.151±2.331 | 81.165±3.150 | 2.613 | (0.089) |
