# Supplementary material for: The Role of Vesicular Glutamate Transporter Type 3 in Social Behavior, with a Focus on the Median Raphe Region
Source: eNeuro. 2024 Jun 3;11(6):ENEURO.0332-23.2024. doi: 10.1523/ENEURO.0332-23.2024 (PMC11154661; doi:10.1523/ENEURO.0332-23.2024)
Supplement: Figure 3-9 — Results of resident intruder test – VGluT3-Cre animals. VGluT3-Cre: Degree of freedom (df) for the one-way ANOVA (all parameters) is (2,31). Data are expressed in mean ± SEM. WT: wild-type; KO: knock-out. == p < 0.01 vs control; @ p < 0.05 vs excitatory. Download Figure 3-9, DOCX file. [file eneuro-11-ENEURO.0332-23.2024-s013.docx]

**Extended Data Table to Figure 3-9. Results of resident intruder test – VGluT3-Cre animals.**

| **DREADD type** | | **Control (N=8** | **Excitatory (N=13)** | **Inhibitory (N=14)** | **F-value** | **p-value** |
| --- | --- | --- | --- | --- | --- | --- |
| **Frequency** | **Social behaviour** | 56.000± 3.546 | 49.692± 3.921 | 39.786$\pm$ 1.962 **== @** | 5.938 | 0.006 |
|  | **Aggressive behaviour** | 5.000±1.543 | 3.154±0.926 | 3.857$\pm$1.600 | 0.344 | 0.711 |
|  | **Defensive behaviour** | 0.143±0.143 | 0.308±0.175 | 0.857$\pm$0.404 | 1.386 | 0.265 |
|  | **‘Other’ behaviour** | 54.571± 2.877 | 49.000± 3.718 | 38.714$\pm$ 2.879 **== @** | 5.308 | 0.010 |
| **Time (%)** | **Social behaviour** | 29.479±2.909 | 35.205±4.572 | 35.068$\pm$4.854 | 0.349 | 0.708 |
|  | **Aggressive behaviour** | 2.761±0.954 | 2.324±1.285 | 2.930$\pm$1.790 | 0.045 | 0.956 |
|  | **Defensive behaviour** | 0.076±0.076 | 0.100±0.069 | 0.681$\pm$0.468 | 1.085 | 0.350 |
|  | **‘Other’ behaviour** | 67.686±2.420 | 62.361±5.288 | 61.322$\pm$5.807 | 0.286 | 0.753 |
