# Supplementary material for: The Role of Vesicular Glutamate Transporter Type 3 in Social Behavior, with a Focus on the Median Raphe Region
Source: eNeuro. 2024 Jun 3;11(6):ENEURO.0332-23.2024. doi: 10.1523/ENEURO.0332-23.2024 (PMC11154661; doi:10.1523/ENEURO.0332-23.2024)
Supplement: Figure 4-1 — Results of social discrimination test – VGluT3 WT-KO animals. Degree of freedom (df) for the two-sample t-test (frequency and time [%] of ‘other’ behaviour; SI) is 18. Degree of freedom in the repeated-measures ANOVA (frequency and time [%] of ‘old’ vs ‘right’ mouse) is (1,18) for all effects. Marginal effects are in brackets (). Data are expressed in mean ± SEM. WT: wild-type; KO: knock-out; SD: social discrimination index. * p < 0.05 vs WT; # p < 0.05 vs cage; $ p < 0.05 vs random 0. Download Figure 4-1, DOCX file. [file eneuro-11-ENEURO.0332-23.2024-s014.docx]

**Extended Data Table to Figure 4-1. Results of social discrimination test – VGluT3 WT-KO animals.**

| **Genotype** | | **WT (N=10)** | **KO (N=11)** | **F- or t-value** | **p-value** |
| --- | --- | --- | --- | --- | --- |
| **Frequency** | **‘Old’ mouse** | 16.333±2.333 | 18.727±2.632 | Genotype:  0.394  Choice:  0.604  Genotype$\times$Choice:  0.147 | 0.538  0.447  0.706 |
|  | **’New’ mouse** | 15.778±1.535 | 17.091±2.302 |  |  |
|  | **‘Other’ behaviour** | 32.111±3.510 | 34.636±4.012 | -0.462 | 0.649 |
| **Time (%)** | **‘Old’ mouse** | 10.232±2.059 | 18.239±2.608***** | Genotype:  3.142  Choice:  1.163  Genotype$\times$Choice:  4.281 | (0.093)  0.295  0.053 |
|  | **’New’ mouse** | 11.806±2.015 | 13.237±1.825**#** |  |  |
|  | **‘Other’ behaviour** | 77.962±3.675 | 68.524±3.757 | 1.773 | (0.093) |
| **SD** | | 10.945±7.069 | -19.329±8.217**$** | 2.503 | 0.023 |
