# Supplementary material for: The Role of Vesicular Glutamate Transporter Type 3 in Social Behavior, with a Focus on the Median Raphe Region
Source: eNeuro. 2024 Jun 3;11(6):ENEURO.0332-23.2024. doi: 10.1523/ENEURO.0332-23.2024 (PMC11154661; doi:10.1523/ENEURO.0332-23.2024)
Supplement: Figure 4-2 — Results of Y-maze test – VGluT3 WT-KO animals. Degree of freedom (df) for the two-sample t-test for locomotion is 19, for alteration is 16. Data are expressed in mean ± SEM. WT: wild-type; KO: knock-out. $$ p < 0.01 vs random 50. Download Figure 4-2, DOCX file. [file eneuro-11-ENEURO.0332-23.2024-s015.docx]

**Extended Data Table to Figure 4-2. Results of Y-maze test – VGluT3 WT-KO animals.**

| **Genotype** | **WT (N=10)** | **KO**  **(N=11)** | **t-value** | **p-value** |
| --- | --- | --- | --- | --- |
| **Locomotion** | 18.200$\pm$3.269 | 19.818$\pm$3.173 | -0.355 | 0.726 |
| **Spontaneous alteration** | 69.364$\pm$1.278  $$ | 64.451$\pm$4.005  $$ | 0.949 | 0.356 |
