# Supplementary material for: The Role of Vesicular Glutamate Transporter Type 3 in Social Behavior, with a Focus on the Median Raphe Region
Source: eNeuro. 2024 Jun 3;11(6):ENEURO.0332-23.2024. doi: 10.1523/ENEURO.0332-23.2024 (PMC11154661; doi:10.1523/ENEURO.0332-23.2024)
Supplement: Figure 4-3 — Results of social discrimination test – VGluT3-Cre animals. Degree of freedom (df) for the one-way ANOVA (frequency and time [%] of ‘other’ behaviour) is (2,32). Degree of freedom in the repeated-measures ANOVA (frequency and time [%] of mouse vs cage) is (2,32) for the effect of manipulation and manipulation × choice interaction, while (1,32) for the effect of choice. Marginal effects are in brackets (). Data are expressed in mean ± SEM. SD: social discrimination index. # p < 0.05 vs cage; == p < 0.01 vs control; @@ p < 0.01 vs excitatory. $ p < 0.05 vs random 0. Download Figure 4-3, DOCX file. [file eneuro-11-ENEURO.0332-23.2024-s016.docx]

**Extended Data Table to Figure 4-3. Results of social discrimination test – VGluT3-Cre animals.**

| **DREADD type** | | **Control (N=8)** | **Excitatory (N=12)** | **Inhibitory (N=15)** | **F- or t-value** | **p-value** |
| --- | --- | --- | --- | --- | --- | --- |
| **Frequency** | **‘Old’ mouse** | 21.125± 1.959 | 20.917± 1.773 | 24.600$\pm$ 1.621 | Manipulation:  3.336  Choice:  0.021  Manipulation$\times$Choice:  0.834 | 0.048  0.886  0.443 |
|  | **’New’ mouse** | 19.000± 2.179 | 22.833± 1.429 | 25.333$\pm$ 1.379 |  |  |
|  | **‘Other’ behaviour** | 40.625± 3.505 | 44.083± 2.395 | 48.933$\pm$ 2.150 | 2.586 | (0.091) |
| **Time (%)** | **‘Old’ mouse** | 9.113± 0.854 | 9.800± 1.148 | 14.832$\pm$ 1.319 | Manipulation:  7.070  Choice:  1.631  Manipulation$\times$Choice:  0.054 | 0.003  0.211  0.948 |
|  | **’New’ mouse** | 9.825± 1.998 | 11.267± 1.077 | 16.045$\pm$ 1.663 |  |  |
|  | **‘Other’ behaviour** | 79.813± 2.688 | 77.550± 1.926 | 68.050$\pm$ 2.620  **== @@** | 6.526 | 0.004 |
| **SD** | | -1.394$\pm$ 7.819 | 7.801± 6.172 | 1.420± 5.198 | 0.530 | 0.593 |
