# Supplementary material for: The Role of Vesicular Glutamate Transporter Type 3 in Social Behavior, with a Focus on the Median Raphe Region
Source: eNeuro. 2024 Jun 3;11(6):ENEURO.0332-23.2024. doi: 10.1523/ENEURO.0332-23.2024 (PMC11154661; doi:10.1523/ENEURO.0332-23.2024)
Supplement: Figure 4-4 — Results of Y-maze test – VGluT3-Cre animals. Degree of freedom (df) for the one-way ANOVA for locomotion is (2,32), while for alteration is (2,31). Data are expressed in mean ± SEM. $$ p < 0.01 vs random 50. Download Figure 4-4, DOCX file. [file eneuro-11-ENEURO.0332-23.2024-s017.docx]

**Extended Data Table to Figure 4-4. Results of Y-maze test – VGluT3-Cre animals.**

| **DREADD type** | **Control (N=8)** | **Excitatory (N=13)** | **Inhibitory (N=14)** | **F-value** | **p-value** |
| --- | --- | --- | --- | --- | --- |
| **Locomotion** | 24.750± 2.226 | 21.846± 1.671 | 22.800± 1.607 | 0.724 | 0.492 |
| **Spontaneous alteration** | 58.371± 5.217 | 63.624± 2.841 **$$** | 65.240± 1.798 **$$** | 1.140 | 0.333 |
