# Supplementary material for: The Role of Vesicular Glutamate Transporter Type 3 in Social Behavior, with a Focus on the Median Raphe Region
Source: eNeuro. 2024 Jun 3;11(6):ENEURO.0332-23.2024. doi: 10.1523/ENEURO.0332-23.2024 (PMC11154661; doi:10.1523/ENEURO.0332-23.2024)
Supplement: Figure 5-1 — Results of c-Fos positive cell counting – VGluT3 WT-KO animals. VGluT3 KO mice showed decreased activity after social test in their anterior cingulate, infralimbic cortex and medial septum. Degree of freedom (df) for the two-sample t-test is 11. Benjamini-Hochberg (FDR, false discovery rate) post-hoc correction was used, thus a p-value was considered significant from 0.016. Data are expressed in mean ± SEM. WT: wild-type; KO: knock-out. * p < 0.05, ** p < 0.01 vs WT Download Figure 5-1, DOCX file. [file eneuro-11-ENEURO.0332-23.2024-s018.docx]

**Extended Data Table to Figure 5-1. Results of c-Fos positive cell counting – VGluT3 WT-KO animals.**

| **Genotype** | | **WT  (N=5)** | **KO (N=8)** | **t-value** | **p-value** |
| --- | --- | --- | --- | --- | --- |
| **Prefrontal cortex** | **Prelimbic cortex** | 51.583$\pm$7.440 | 35.000$\pm$7.003 | 1.667 | 0.124 |
|  | **Anterior cingulate** | 64.083$\pm$6.052 | 38.729$\pm$7.457 | 2.383 | 0.036 |
|  | **Infralimbic cortex** | 51.000$\pm$6.533 | 28.104$\pm$3.483****** | 3.402 | 0.006 |
| **Medial septum** | | 36.133$\pm$6.455 | 13.125$\pm$1.805****** | 4.199 | 0.001 |
| **Hippocampus** | **CA1 region** | 33.714$\pm$13.140 | 8.440$\pm$3.354 | 1.992 | 0.072 |
|  | **CA3 region** | 35.661$\pm$12.465 | 16.964$\pm$3.203 | 1.552 | 0.149 |
|  | **Dentate gyrus** | 24.889$\pm$13.356 | 13.345$\pm$5.876 | 2.076 | 0.062 |
| **Median raphe region** | | 17.843$\pm$2.976 | 24.583$\pm$5.741 | -0.872 | 0.402 |
