# Supplementary material for: The Role of Vesicular Glutamate Transporter Type 3 in Social Behavior, with a Focus on the Median Raphe Region
Source: eNeuro. 2024 Jun 3;11(6):ENEURO.0332-23.2024. doi: 10.1523/ENEURO.0332-23.2024 (PMC11154661; doi:10.1523/ENEURO.0332-23.2024)
Supplement: Figure 5-2 — Results of c-Fos positive cell counting – VGluT3-Cre animals. The excitatory group showed increased neuronal activity only in the viral injected target area, the median raphe region. Degree of freedom (df) for the two-sample t-test is 7. Benjamini-Hochberg (FDR, false discovery rate) post-hoc correction was used, thus a p-value was considered significant from 0.008. Data are expressed in mean ± SEM. ## p < 0.01 vs Control. Download Figure 5-2, DOCX file. [file eneuro-11-ENEURO.0332-23.2024-s019.docx]

**Extended Data Table to Figure 5-2. Results of c-Fos positive cell counting – VGluT3-Cre animals.**

| **DREADD type** | | **Control  (N=6)** | **Excitatory (N=3)** | **t-value** | **p-value** |
| --- | --- | --- | --- | --- | --- |
| **Prefrontal cortex** | **Prelimbic cortex** | 10.278$\pm$1.737 | 9.033$\pm$1.889 | 0.4402 | 0.673 |
|  | **Infralimbic cortex** | 10.667$\pm$1.907 | 17.133$\pm$3.135 | -1.867 | 0.104 |
| **Medial septum** | | 1.500$\pm$0.624 | 1.333$\pm$0.192 | 0.197 | 0.849 |
| **Hippocampus** | **CA1 region** | 11.472$\pm$1.327 | 13.833$\pm$0.928 | -1.159 | 0.284 |
|  | **CA3 region** | 9.250$\pm$0.846 | 10.944$\pm$1.011 | -1.206 | 0.267 |
|  | **Dentate gyrus** | 24.694$\pm$1.057 | 26.389$\pm$2.237 | -0.795 | 0.452 |
| **Median raphe region** | | 2.389$\pm$1.236 | 41.333$\pm$2.848**##** | -14.987 | <0.001 |
