# Supplementary material for: The Role of Vesicular Glutamate Transporter Type 3 in Social Behavior, with a Focus on the Median Raphe Region
Source: eNeuro. 2024 Jun 3;11(6):ENEURO.0332-23.2024. doi: 10.1523/ENEURO.0332-23.2024 (PMC11154661; doi:10.1523/ENEURO.0332-23.2024)
Supplement: Figure 5-3 — Subject information for the RT-PCR measurements in the brainstem of humans. Download Figure 5-3, DOCX file. [file eneuro-11-ENEURO.0332-23.2024-s020.docx]

| **Extended Data Table to Figure 5-3. Subject information for the RT-PCR measurements in the brainstem of humans.** | | | | |
| --- | --- | --- | --- | --- |
| **Brain No.** | **Biological sex** | **Age** | **Post mortem delay** | **Cause of death** |
| **#165** | female | 88 | 2.5 h | cardiac and respiratory insufficiency |
| **#186** | female | 56 | 5 h | myocardial infarction |
| **#211** | female | 56 | 6 h | cardiorespiratory insufficiency |
| **#216** | male | 53 | 5 h | pulmonary embolism |
| **#227** | male | 55 | 6 h | acute myocardial infarction |
| **#228** | male | 27 | 8 h | pneumonia |
| **#231** | female | 55 | 5 h | bronchopneumonia, cardiorespiratory insufficiency |
| **#242** | male | 50 | 5.5 h | stroke, brain haemorrhage |
| **#244** | male | 53 | 2 h | acute myocardial infarction |
| **#256** | male | 67 | 10 h | pancreas cancer |
| **#266** | male | 61 | 8 h | stroke |
| **#267** | female | 91 | 8 h | stroke |
| **#272** | female | 75 | 10 h | stroke |
| **#273** | male | 64 | 10 h | stroke |
| **#282** | female | 94 | 4 h | stroke, hypertension |
