# Supplementary material for: The Role of Vesicular Glutamate Transporter Type 3 in Social Behavior, with a Focus on the Median Raphe Region
Source: eNeuro. 2024 Jun 3;11(6):ENEURO.0332-23.2024. doi: 10.1523/ENEURO.0332-23.2024 (PMC11154661; doi:10.1523/ENEURO.0332-23.2024)
Supplement: Figure 5-4 — RT-PCR results of human samples. Four major brainstem nuclei were investigated, out of which all showed VGluT3 expression on an mRNA level. The results show averaged and normalized CT values. VGluT3: vesicular glutamate transporter type 3. Download Figure 5-4, DOCX file. [file eneuro-11-ENEURO.0332-23.2024-s021.docx]

**Extended Data Table to Figure 5-4. RT-PCR results of human samples.**

| **Brain area** | **Donor** | **housekeeping genes** | **VGluT3** | **Relative expression** |
| --- | --- | --- | --- | --- |
| **Lateral parabrachial**  **nucleus** | **#227** | 15.706 | 29.53 | 6.90E-05 |
|  | **#256** | 15.563 | 28.6 | 1.19E-04 |
|  | **#266** | 17.972 | 0 | 0.00E+00 |
| **Pontine reticular formation** | **#165** | 15.935 | 28.54 | 1.60E-04 |
|  | **#186** | 16.815 | 29.92 | 1.14E-04 |
|  | **#211** | 15.266 | 28.05 | 1.42E-04 |
|  | **#216** | 15.576 | 29.04 | 8.85E-05 |
|  | **#227** | 14.385 | 27.49 | 1.13E-04 |
|  | **#228** | 15.533 | 30.18 | 3.90E-05 |
|  | **#231** | 18.547 | 33.63 | 2.88E-05 |
|  | **#242** | 18.836 | 31.97 | 1.11E-04 |
|  | **#244** | 19.397 | 31.79 | 1.86E-04 |
|  | **#256** | 15.115 | 28.51 | 9.28E-05 |
| **Pontine raphe nucleus** | **#211** | 14.808 | 23.49 | 4.66E-03 |
|  | **#216** | 14.339 | 26.98 | 1.31E-04 |
|  | **#227** | 14.495 | 27.06 | 2.58E-04 |
|  | **#231** | 18.203 | 32.83 | 5.40E-06 |
|  | **#244** | 14.326 | 25.81 | 4.85E-04 |
|  | **#256** | 15.715 | 28.43 | 8.17E-05 |
| **Midbrain raphe** | **#186** | 17.547 | 30.84 | 1.54E-05 |
|  | **#244** | 15.515 | 26.66 | 5.04E-04 |
|  | **#266** | 14.773 | 28.93 | 9.47E-05 |
